# Shelterin TPP1 Promotes Hair Regeneration Through Activating Bulge Hair Follicle Stem Cells

**DOI:** 10.1111/acel.70692

**Published:** 2026-08-28

**Authors:** Lihui Wang, Bihan Zhao, Jiahui Yang, Ying Liang, Yitian Yao, Xiao Xiao, Long Yang, Jinghao Xia, Haoze Li, Ling Gao, Jun Zhang, Tianci Su, Hao Xu, Jiacheng Zhang, Hongyan Wang, Jun Liu, Xiaojing Hong, Jun‐Ping Liu

**Affiliations:** ^1^ Institute of Ageing Research Hangzhou Normal University, School of Basic Medical Sciences Hangzhou Zhejiang China; ^2^ Postgraduate Program on Ageing Biology Hangzhou Normal University, School of Basic Medical Sciences Hangzhou Zhejiang China; ^3^ Hangzhou Duanli Biotechnology Company Limited Hangzhou Zhejiang China; ^4^ Department of Immunology and Pathology, Central Clinical School, Faculty of Medicine Monash University Prahran Victoria Australia

**Keywords:** bulge hair follicle stem cells (bHFSCs), hair growth cycle, hair regeneration, shelterin, telomerase, telomere, telomere capping, telomere elongation

## Abstract

Telomere shortening drives hair follicle senescence, yet successful reversal of this aging process has remained elusive. Here, we report that stem cell overexpression of *Acd*, the gene encoding TPP1 (telomere protection protein 1)—a shelterin component that recruits telomerase—accelerates hair regeneration in mice. Stabilization of TPP1 protein via topical application of TELODIN, a synthetic octapeptide that inhibits TPP1 degradation, similarly promotes hair regeneration. Mechanistically, increased TPP1 mobilizes bulge hair follicle stem cells (bHFSCs) by coordinating telomere capping and telomerase recruitment. By facilitating the synchronization and transition of hair follicles from the quiescent telogen phase to the proliferative anagen phase, TPP1 drives bHFSCs proliferation, differentiation, and migration. These findings identify TPP1 as a master regulator of hair regeneration through telomere capping and elongation, and suggest that topical TELODIN represents a novel therapeutic strategy for advancing hair growth. This work establishes a therapeutic framework for telomere‐targeted interventions against alopecia.

## Graphical abstract

Elongation of telomeres by increasing TPP1, through genetic and pharmacological approaches, promotes hair follicle bulge stem cell mobilization and migration, leading to accelerated telogen‐to‐anagen transition and hair regeneration. This work identifies TPP1 as a master regulator and therapeutic target for alopecia.

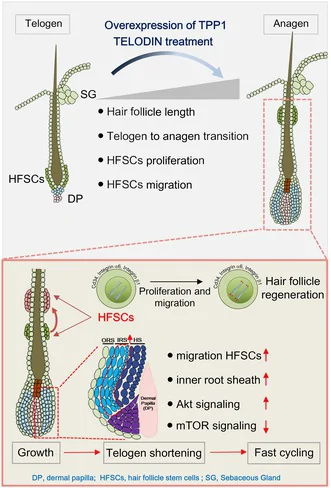

**Abbreviations**
Acd: adrenocortical dysplasia
BC: basal cells
bHFSCs: bulge hair follicle stem cells (bHFSCs)
DEGs: differentially expressed genes
EMT: epithelial‐mesenchymal transition
FACS: fluorescence‐activated cell sorting
FCs: fibroblast cells
FISH: fluorescence in situ hybridization
GCs: germinative layer cells
GSEA: gene set enrichment analysis
HF: hair follicle
HS1: hair shaft 1
HS2: hair shaft 2
IF: immunofluorescence
IRS: inner root sheath
LCs: Langerhans cells
MC: mitotic cells
OB: oligonucleotide/oligosaccharide binding
PFA: paraformaldehyde
qPCR: quantitative real‐time polymerase chain reaction
SC: sebaceous gland cells
SM: smooth muscle
TCs: T cells
TEL patch: TPP1 glutamate (E) and leucine (L)‐rich patch
TELODIN: telomere‐dysfunction inhibitor
TERT: telomerase reverse transcriptase
TIFs: telomere dysfunction‐induced foci
TPP1: telomere protection protein 1
UMAP: uniform manifold approximation and projection

## 1 Introduction

Telomeres—the DNA–protein complexes at chromosome ends—protect chromosomal DNA from damage and maintain genome stability (Blackburn 2001; de Lange 2026). Telomeres are lengthened, which counteracts aging‐related attrition, and is mediated by telomerase activity in stem cells through shelterin‐mediated recruitment of the enzyme (de Lange 2026; Marion et al. 2009; Takahashi et al. 2007). The critical role of telomere maintenance underpins cell replicative potential (Patel et al. 2016; Tomás‐Loba et al. 2008) during stem cell proliferation, differentiation, and function (Pucci et al. 2013). Dysfunctional telomere maintenance predisposes individuals to numerous age‐associated diseases, whereas proper maintenance is tightly linked to tissue repair and regeneration (Armanios 2022; Mosteiro et al. 2016; Razdan et al. 2018). While many organs decline functionally and structurally due to telomere shortening during aging (Campisi 2005), it remains unclear whether maintaining telomere length directly promotes regeneration by expanding regenerative cell populations, or what the underlying mechanisms are (Mosteiro et al. 2016; Razdan et al. 2018).

A strategy to counteract stress‐induced acceleration of telomere attrition is to generate telomerase primers and recruit the enzyme complex to telomeric chromatin via shelterin (de Lange 2026). The shelterin component TPP1 (telomere protection protein 1, also known as TINT1/PTOP/PIP1) recruits telomerase through a direct interaction between the glutamate (E) and leucine (L)‐rich patch (TEL‐patch) in its oligonucleotide/oligosaccharide‐binding (OB) fold and the catalytic TERT subunit of telomerase (Abreu et al. 2010; de Lange 2026; Wang et al. 2007). Epidermal Tpp1 deficiency results in severe skin hyperpigmentation and aberrant hair follicle morphogenesis (Tejera et al. 2010). Our previous studies demonstrated that increasing TPP1—either through genetic overexpression of *Acd* (encoding TPP1) or via protein stabilization using an octapeptide—alleviates pulmonary alveolar stem cell senescence and respiratory dysfunction, thereby preventing pulmonary fibrosis (Wang et al. 2020). However, whether TPP1 stabilization promotes hair follicular stem cell regeneration remains to be demonstrated.

Premature aging of hair follicle (HF) leads to hair loss (alopecia), a condition that adversely affects human well‐being globally. Hair follicle dysregulation involves genetic, hormonal, metabolic, and immune modulatory factors (Cohen et al. 2024; Oh et al. 2016; Shin et al. 2020; Yang et al. 2023). Deficiencies in the telomere maintenance program have been shown to negatively impact both hair growth and post‐injury regeneration (Sarin et al. 2005; Siegl‐Cachedenier et al. 2007). As intricate epidermal invaginations, HFs are susceptible to aging phenotypes such as graying and hair loss due to stem cell senescence (Matsumura et al. 2016). HFs are maintained by a pool of hair follicle stem cells (HFSCs), which originate from the upper placode during early morphogenesis and later reside in a specialized niche called the bulge (Morita et al. 2021). In mice, although HF development begins postnatally (P0), the definitive bulge niche architecture only becomes distinct around 3 weeks of age (P20–P21), coinciding with full hair coat formation (Cotsarelis 2006; Joulai Veijouye et al. 2017). At this stage, bulge cells upregulate specific stem cell markers, including Cd34 and a keratin 15‐LacZ reporter (Blanpain et al. 2004; Trempus et al. 2003). Bulge hair follicle stem cells (bHFSCs), defined by key markers such as Sox9, Lhx2, Nfatc1, Tcf3, and Lgr5 (Horsley et al. 2008; Morris et al. 2004; Nguyen et al. 2006; Rhee et al. 2006; Tumbar et al. 2004), are capable of generating all epithelial cell types of the follicle and hair shaft during the hair cycle (Morris et al. 2004). Throughout life, HFs undergo cyclic phases: rapid growth (anagen), regression (catagen), and quiescence (telogen) (Muller‐Rover et al. 2001; Yang et al. 2023). Telogen HFs, in particular, exhibit remarkable regenerative plasticity (Rosen et al. 2020; Segal‐Bendirdjian and Geli 2019). However, stem cell exhaustion drives hair follicle aging, culminating in failed hair regeneration (Zhang et al. 2021).

In this study, we employed genetic and pharmacological approaches to investigate the effect of TPP1 on hair growth in mouse models. We demonstrate that increasing TPP1 robustly accelerates hair regeneration. Mechanistically, TPP1 activates HFSCs by preventing telomeric DNA damage. Using single‐cell RNA sequencing, we revealed that the octapeptide telomere‐dysfunction inhibitor (TELODIN) stimulates bHFSCs proliferation, migration, and differentiation during hair regeneration. These findings indicate that TPP1 is a key regulator of hair follicle cycling, and that TELODIN‐mediated TPP1 stabilization represents a promising approach to promote hair regeneration by mobilizing HFSCs through telomere maintenance.

## 2 Results

### 2.1 TPP1 Promotes HFSCs Activation and Hair Regeneration

**FIGURE 1:**
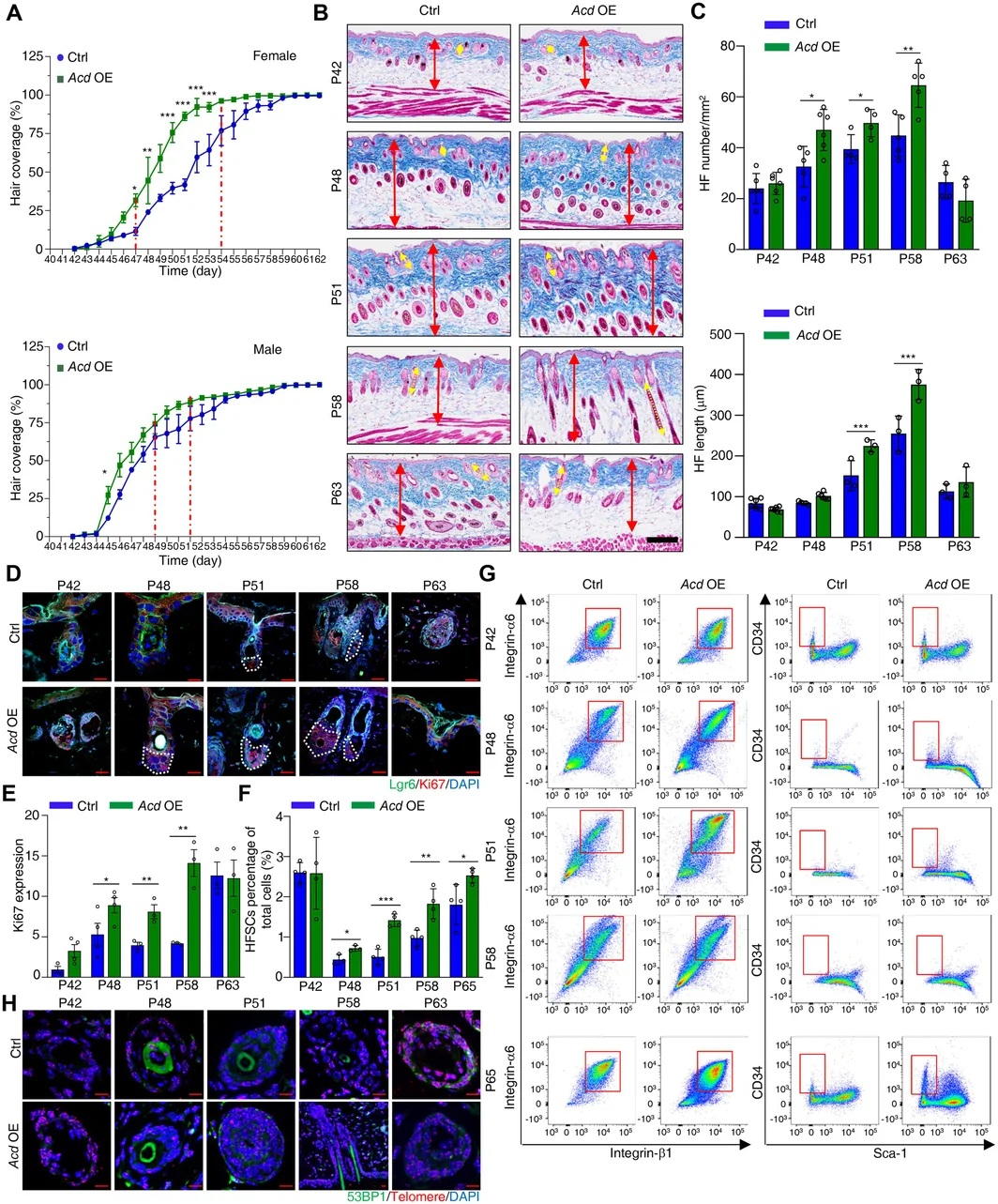
TPP1 induces HFSCs and promotes mouse hair regeneration. (A) Hair regrowth assay was performed on WT control and *Acd*‐overexpressed mice. The dorsal skin was shaved at P42. The hair coverage percentage was quantified from six mice per group. (B) Representative Masson's trichrome staining images. Yellow double‐headed arrows denote HF length and red arrows indicate dermal thickness. Scale bar = 200 μm. (C) Quantitative analysis of HF number (number/mm) and hair length at P42, 48, P51, P58, and P63. (D) Immunofluorescence staining of Ki67 and Lgr6 in WT control and *Acd*‐overexpressed mice at indicated time points. Dotted lines outline anagen hair bulbs. Scale bar = 100 μm. (E) qPCR was performed to detect the expression of Ki67. (F, G) bHFSCs were isolated from mouse dorsal skin by flow cytometry using surface markers (Itga6^high^, Itgb1^+^, Sca‐1^−^, and Cd34^+^) at P42, P48, P51, P58, and P65. The proportion of bHFSCs among total cells was quantified in four mice per group. (H) IF‐FISH analysis with 53BP1 antibody (Green) and telomere probe (Red) assessed telomere DNA damage in paraffin‐embedded skin tissues. Scale bar = 25 μm. All data represent mean ± SEM. *p < 0.05; **p < 0.01; ***p < 0.001.

To investigate the role of the TPP1‐encoding *Acd* gene in hair regeneration, we generated a floxed *Acd* allele and overexpressed TPP1 in keratinocytes using *Krt14*‐Cre, creating *mAcd* ^*flox/flox;Krt14‐Cre*^ mice with cell type‐specific TPP1 overexpression (OE; Figure S1). Remarkably, the hair coat of *mAcd* ^*flox/flox;Krt14‐Cre*^ mice regenerated earlier than that of control mice after shaving, as evidenced by dorsal skin color changes (Figure S2A). In female mice, skin pigmentation—indicative of anagen onset—began at postnatal day P48–49 in 65% of *mAcd* ^*flox/flox;Krt14‐Cre*^ mice, compared to P51–52 in controls (Figure S2A). Furthermore, hair growth rates were significantly higher in female *mAcd* ^*flox/flox;Krt14‐Cre*^ mice between P47 and P54 (Figure 1A).

Consistent with enhanced hair growth, histological analysis revealed that HF number, HF length, and dermal thickness increased starting at P48 and plateaued between P51 and P58 in *mAcd* ^*flox/flox;Krt14‐Cre*^ mice (Figure 1B,C and Figure S2B). Notably, HFs in *mAcd* ^*flox/flox;Krt14‐Cre*^ mice entered the anagen phase earlier than those in controls (Figure 1B). Following this early entry, HFs subsequently underwent miniaturization, and dermal thickness decreased by P63 (Figure 1B), collectively indicating an accelerated HF cycle. Thus, elevated TPP1 levels promote hair regeneration by accelerating HF cycle progression. Using Lgr6^+^ cells—a biomarker expressed in HFSCs between the hair germ and interfollicular epidermis (Snippert et al. 2010)—we detected Lgr6^+^ cells with low Ki67 expression in both control and *m* *Acd* ^*flox/flox;Krt14‐Cre*^ mice at P42, consistent with the telogen phase. By P48, *m* *Acd* ^*flox/flox;Krt14‐Cre*^ mice exhibited increased Lgr6^+^ cell numbers, elevated Ki67 expression, and active hair growth, indicating anagen entry, whereas controls remained in telogen (Figure 1D). By P58, the expression of both Lgr6 and Ki67 had plateaued in *m* *Acd* ^*flox/flox;Krt14‐Cre*^ mice, with Lgr6 localized to the upper HF region (Figure 1D). Furthermore, *Acd* overexpression consistently upregulated Ki67 expression (Figure 1E). Together, these results demonstrate that TPP1 enhances HF regeneration by stimulating HFSCs proliferation.

To investigate the role of *Acd* overexpression in HFSCs during hair growth, we isolated HFSCs (Itga6^high^/Itgb1^+^/Sca‐1^−^/Cd34^+^) from mouse dorsal skin using fluorescence‐activated cell sorting (FACS) as previously described (Morris et al. 2004). Compared to controls, the HFSCs percentage in *m* *Acd* ^*flox/flox;Krt14‐Cre*^ mice was 251%, 188%, 185%, and 129% of control values at P48, P51, P58, and P65, respectively (Figure 1F,G). The identity of sorted cells was validated by immunofluorescence and quantitative real‐time polymerase chain reaction (qPCR) (Figure S2C,D,D′). Moreover, both the HFSCs marker Integrin α6 and TPP1 itself were expressed at higher levels in *Acd*‐overexpressing HFSCs (Figure S2E,E′). To assess TPP1's role in telomere maintenance, we compared telomere dysfunction after shaving. Consistent with its function, TPP1 overexpression significantly reduced telomeric DNA damage—as measured by telomere dysfunction‐induced foci (TIFs)—in HFs (Figure 1H and Figure S2F) and in sorted HFSCs from mutant mice compared to controls (Figure S2G,H). Collectively, these results demonstrate that keratinocyte‐specific TPP1 overexpression expands the HFSCs pool, upregulates HFSCs markers, and attenuates telomeric DNA damage within these cells.

### 2.2 TELODIN Stabilizes TPP1 and Promotes Telogen‐to‐Anagen Transition in Murine Hair Follicles

We next investigated the role of TELODIN in hair growth. TELODIN is an 8‐mer peptide that specifically inhibits stress‐induced TPP1 degradation by the E3 ubiquitin ligase FBW7 (Wang et al. 2020, 2021). After shaving, we topically applied TELODIN daily to the dorsal skin; α‐ketoglutarate (α‐KG), a known promoter of hair growth, served as a positive control (Chai et al. 2019). As shown in Figure S3A, TELODIN significantly enhanced hair growth. Skin pigmentation, indicative of anagen onset, appeared at P49 with TELODIN treatment, which was earlier than with α‐KG (P53) or in untreated controls. H&E staining revealed that TELODIN increased HF numbers, consistent with anagen induction (Figure S3B). Furthermore, TELODIN treatment increased the proliferation of Ki67^+^ cells (Figure S3C). To assess TELODIN's role in subsequent hair regeneration, we analyzed its effects on hair growth at P65, during the late phase of the second HF cycle. Histological analysis showed that TELODIN accelerated secondary HF cycle progression (Figure S3D). Specifically, HFs in TELODIN‐treated mice reached full size, whereas controls remained in telogen (Figure S3E). Consistently, Ki67 expression was higher in TELODIN‐treated HFs at P77 (Figure S3F). These findings demonstrate that TELODIN significantly promotes telogen‐to‐anagen transition in murine dorsal HFs.

**FIGURE 2:**
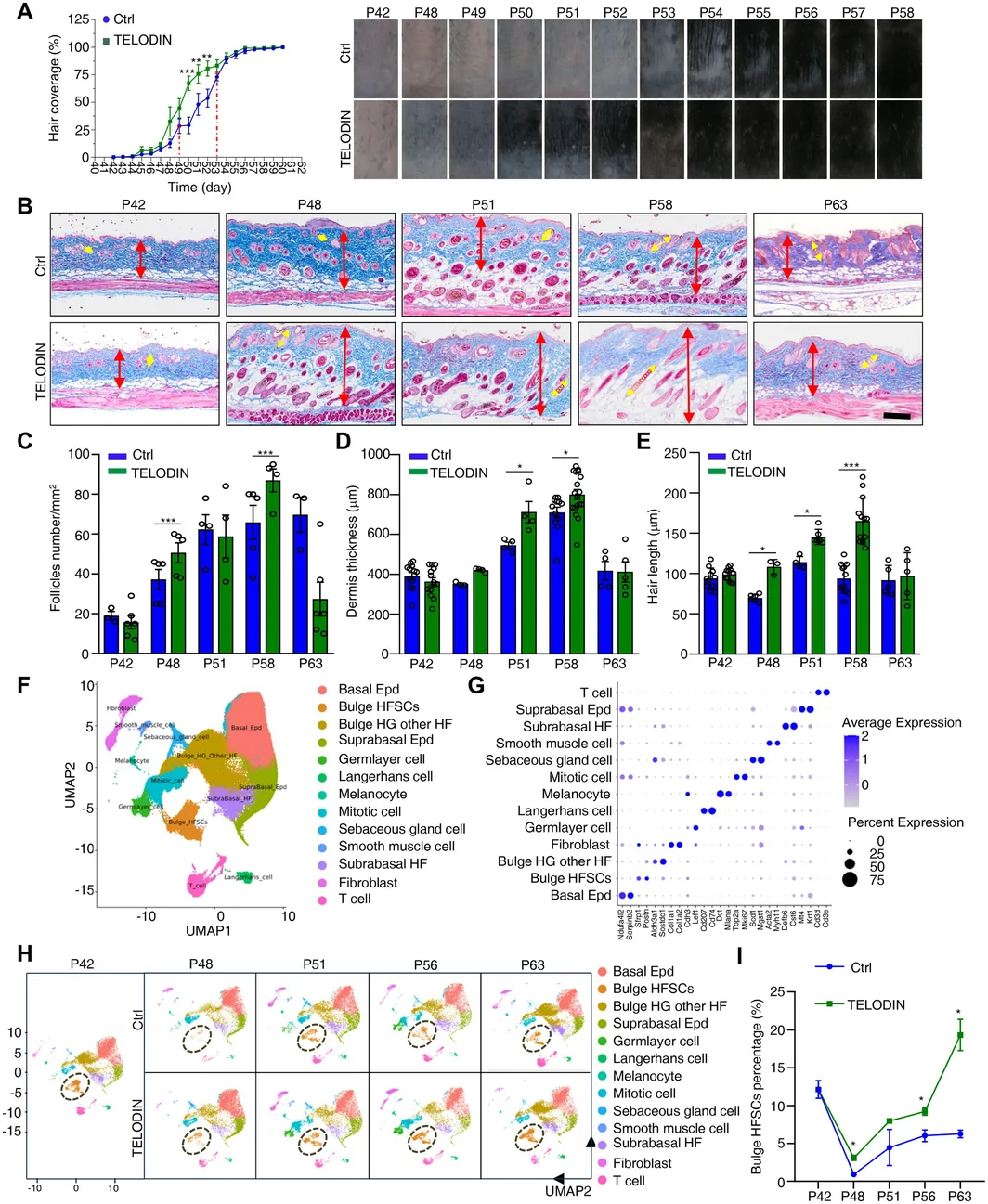
TELODIN promotes telogen‐to‐anagen transition in murine HFs. (A) Hair regrowth assay was performed on male mice from control and TELODIN‐treated groups. Dorsal skin was shaved at P42, followed by daily topical application of either control solution or TELODIN. Hair growth was documented photographically at indicated time points (P40–P62). Hair coverage percentage was quantified (left panel). (B) Histological analysis of dorsal skin collected at P42, P48, P51, P58, and P63 using Masson's trichrome staining. Yellow dashed arrows denote hair follicle length, solid red arrows indicate dermal thickness. Scale bar = 200 μm. (C–E) Quantitative analysis of HF number (number/mm), dermis thickness and hair length at P42, 48, P51, P58, and P63. (F) Single‐cell transcriptome profiling: Uniform manifold approximation and projection (UMAP) visualization of 13 distinct cell populations in mouse skin (cell types annotated at right). (G) Dot plot displaying cell type‐specific marker gene expression. (H) Comparative UMAP analysis of cellular composition between experimental groups. The dashed‐line circle indicates bHFSCs. (I) Quantitative assessment of bHFSCs frequency among total cells. All data represent mean ± SEM. *p < 0.05; **p < 0.01; ***p < 0.001.

Following depilation at P42, TELODIN‐treated mice developed hair follicle growth‐associated skin pigmentation at P48–49, compared to P51–52 in vehicle‐treated controls (Figure 2A and Figure S4A). Histological analysis at early anagen confirmed accelerated anagen entry. Specifically, TELODIN treatment significantly increased HF number (to 136% of control), dermal thickness (120%), and hair length (154%) (Figure 2B–E and Figure S4B–E; *n* = 5–6 mice per group). During P51–P58, both dermal thickness and hair length continued to increase in TELODIN‐treated mice, reaching 113%–131% and 129%–178% of control values, respectively, and peaked prior to catagen onset (Figure 2B–E and Figure S4B*–*E). Taken together, these data demonstrate that TELODIN enhances hair regeneration primarily by accelerating HF cycle progression.

We performed single‐cell RNA sequencing (scRNA‐seq) on skin tissues from TELODIN‐treated and control mice at P42, P48, P51, P56, and P63. After unsupervised clustering using uniform manifold approximation and projection (UMAP) and cell‐type annotation based on established markers (Ge et al. 2020; Zhang et al. 2021; Zhao et al. 2023), we identified 13 distinct cell populations. These included basal cells (BC), bulge HFSCs (bHFSCs), hair germ cells, other HF epithelial cells (bulge/HG/other HF), suprabasal cells, germinative layer cells (GCs), Langerhans cells (LCs), melanocytes, mitotic cells (MCs), sebaceous gland cells (SCs), smooth muscle (SM) cells, fibroblast cells (FCs), and T cells (TCs) (Figure 2F,G). TELODIN treatment markedly increased the proportion of bHFSCs (> 3‐fold), correlating with enhanced hair regeneration (Figure 2H,I).

### 2.3 TELODIN Accelerates Hair Growth Cycle by Stabilizing TPP1 and Preventing Telomere Dysfunction

**FIGURE 3:**
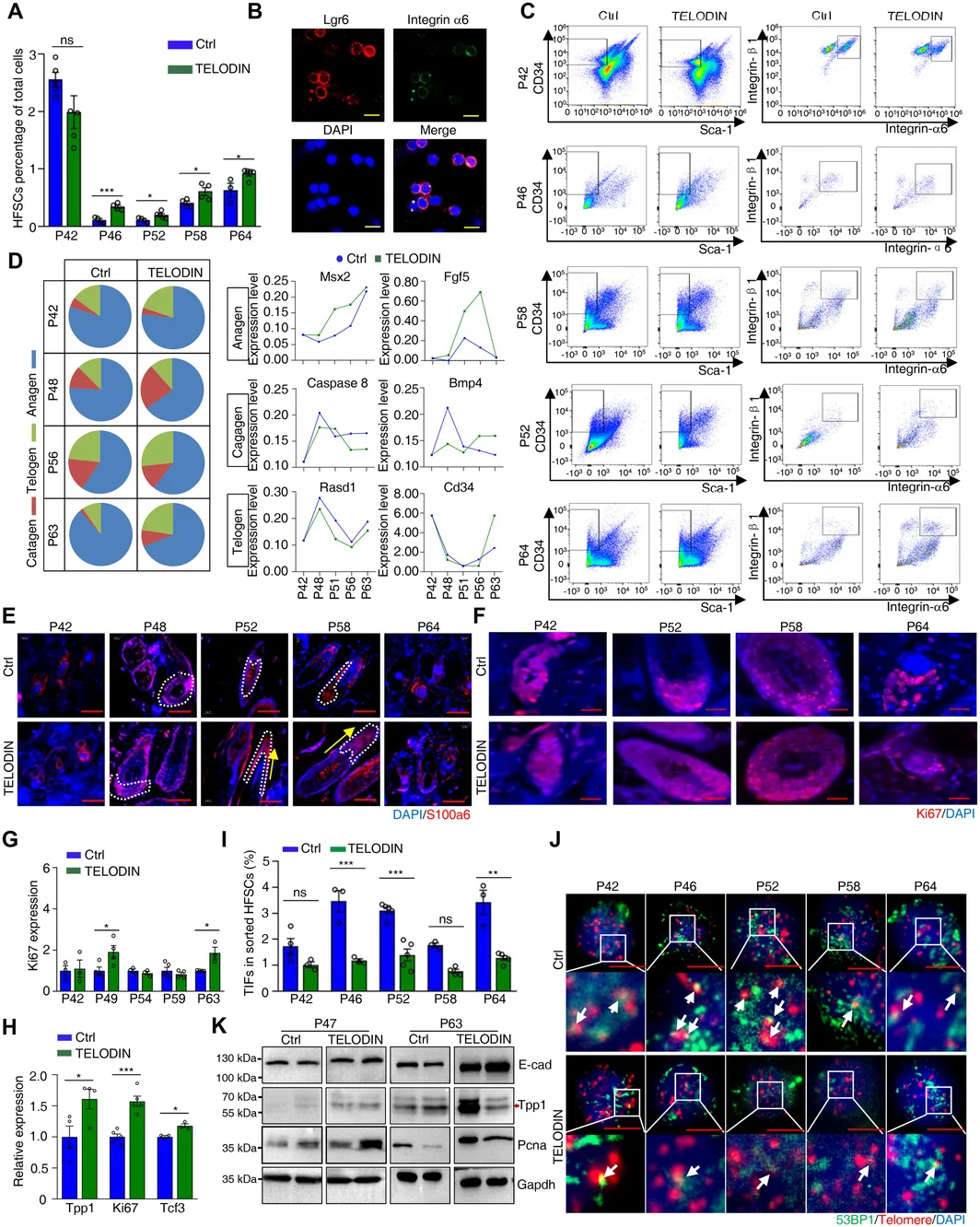
Enhanced bHFSCs promotes cyclic hair regeneration. (A–C) bHFSCs were isolated from mouse dorsal skin by FACS using surface markers (Itga6^high^, Itgb1^+^, Sca‐1^−^, and Cd34^+^) at P42, P46, P52, P58, and P64. The frequency of bHFSCs among total cells was quantified in five mice per group (A, C). Immunofluorescence staining confirmed Lgr6 and Itga6 expression in sorted bHFSCs (B). Scale bar = 10 μm. (D) Hair cycle staging was determined by single‐cell RNA sequencing and quantifying the proportion of follicles in telogen, anagen, and catagen phases. The expression of two representative genes in each phase of the hair follicle was shown. (E) Immunofluorescence microscopy of S100a6 proliferating cells in control and TELODIN‐treated mice. Scale bar = 50 μm. (F) Immunofluorescence microscopy of Ki67 positive stem cell populations in control and TELODIN‐treated mice. Scale bar = 100 μm. (G) qPCR analysis of Ki67 expression in whole skin extracts at indicated time points. (H) qPCR analysis of Ki67, TPP1, and Tcf3 expression in whole skin extracts. (I–J) IF‐FISH analysis with 53BP1 antibody (Green) and telomere probe (Red) assessed telomere DNA damage in sorted HFSCs. Scale bar = 10 μm. (K) Western blot analyzed E‐cadherin, Tpp1, and Pcna expression in skin tissues of control and TELODIN‐treated mice at P47 and P63, with Gapdh as loading control. All data represent mean ± SEM. *p < 0.05; **p < 0.01; ***p < 0.001; ns, no significance.

As determined by FACS of bHFSCs (Itga6^high^/Itgb1^+^/Sca1^–^/Cd34^+^) isolated, the percentage of bHFSCs was significantly increased in TELODIN‐treated mice from P46 to P64 (Figure 3A,C and Figure S5A,B). bHFSCs identity was confirmed by both immunofluorescence (IF) staining for Lgr6 and Integrin α6 (Figure 3B) and qPCR analysis showing elevated expression of a panel of bHFSCs markers (e.g., *Tcf3*, *Tcf4*, *Foxc1*, *Itga6*, *Lgr5*, *S100a6*, *Sox9*, *Krt15*, *Cd34*, *Lhx2*, and *Nfatc1*; Figure S5C). TELODIN significantly upregulated bHFSCs markers in both sexes from P49 to P63 (Figure S6A–E). Analysis of hair cycle stage distribution and the expression dynamics of representative telogen, anagen, and catagen marker genes confirmed that TELODIN treatment significantly accelerated the telogen‐to‐anagen transition (Figure 3D). These results demonstrate that TELODIN promotes hair cycle progression.

We next assessed bHFSCs proliferation over time. Specifically, S100a6—a known bulge and outer root sheath marker (Ito and Kizawa 2001)—showed increased expression in TELODIN‐treated mice at P48 (Figure 3E). In controls, Ki67^+^ bHFSCs were mainly localized to the bulge region, whereas TELODIN treatment induced their distribution throughout the HF by P52 (Figure 3F). HF proliferation peaked at P58 in both groups before declining by P64 (Figure 3F). qPCR analysis confirmed that the mRNA levels of Ki67 and Tcf3 were significantly upregulated in TELODIN‐treated mice (Figure 3G,H). Taken together, these results demonstrate that topical TELODIN accelerates the hair growth cycle by promoting bHFSCs proliferation and their entry into anagen.

Consistent with previous findings in pulmonary alveolar stem cells (Wang et al. 2020), TELODIN enhanced telomere capping, as evidenced by significantly decreased TIFs in HFs at P59 and P63 (Figure S5D,E). Immunofluorescence analysis of sorted bHFSCs confirmed that TELODIN attenuated telomeric DNA damage responses during hair regeneration (Figure 3I,J). TPP1 levels were elevated at P63 (Figure 3K and Figure S5F), and Pcna (a proliferation marker) expression was also significantly upregulated at the same time point (Figure 3K and Figure S5F). These data demonstrate that TELODIN increases telomere capping in HFs through TPP1 stabilization.

### 2.4 TELODIN Does Not Rescue the Hair Regeneration Defect Caused by Telomerase Deficiency in *Tert* Knockout Mice

To determine whether TELODIN promotes hair regeneration by stabilizing TPP1 in a telomerase‐dependent manner, we investigated the effect of TELODIN on hair growth in mice deficient in the telomerase catalytic subunit, Tert. Notably, *Tert* deletion compromised hair growth, consistent with previous studies demonstrating the central role of telomerase in telomere maintenance in hair follicles (Sarin et al. 2005). *Tert* deficiency significantly impaired hair regeneration in both male and female knockout mice (Figure S7A). Compared to controls, *Tert* KO mice exhibited a sustained reduction in hair coverage from P42 to P60. However, treatment with TELODIN failed to accelerate hair growth in *Tert* KO mice (Figure S7B). In addition to the unimproved hair coverage, HF number, length, structure, and dermal thickness remained unresponsive to TELODIN treatment from P42 to P63 (Figure S8A,B). The percentage of bHFSCs was not significantly altered by TELODIN treatment in *Tert*‐deficient mice from P42 to P63 (Figure S8C). Furthermore, TELODIN treatment did not alter telomere length in *Tert* KO mice (Figure S9A,B). Together, these results suggest that functional Tert is indispensable for TELODIN to promote hair regeneration and that TELODIN stimulates hair growth through both telomere capping and elongation via TPP1 stabilization.

### 2.5 Single‐Cell RNA Sequencing Reveals TELODIN‐Stimulated bHFSCs Differentiation and Migration During Hair Regeneration

We performed single‐cell transcriptome profiling on control and TELODIN‐treated groups. In addition to GCs, the number of melanocytes also significantly increased in TELODIN‐treated mice, with distinct gene expression patterns (Figure S10A). TELODIN upregulated genes involved in cell cycle regulation, cell differentiation, extracellular matrix organization, and cell adhesion (Figure S10B). Gene set enrichment analysis (GSEA) revealed TELODIN‐induced enrichment of terms related to myogenesis, epithelial‐mesenchymal transition (EMT), and the Akt signaling pathway (Figure S10C). Moreover, TELODIN activated proliferation‐and migration‐related genes (Figure S10D). GSEA further showed that gene sets related to cell division and telomere maintenance (including the telomerase pathway) were enriched upon TELODIN treatment (Figure S10E), suggesting that TELODIN promotes the hair cycle through telomere maintenance.

**FIGURE 4:**
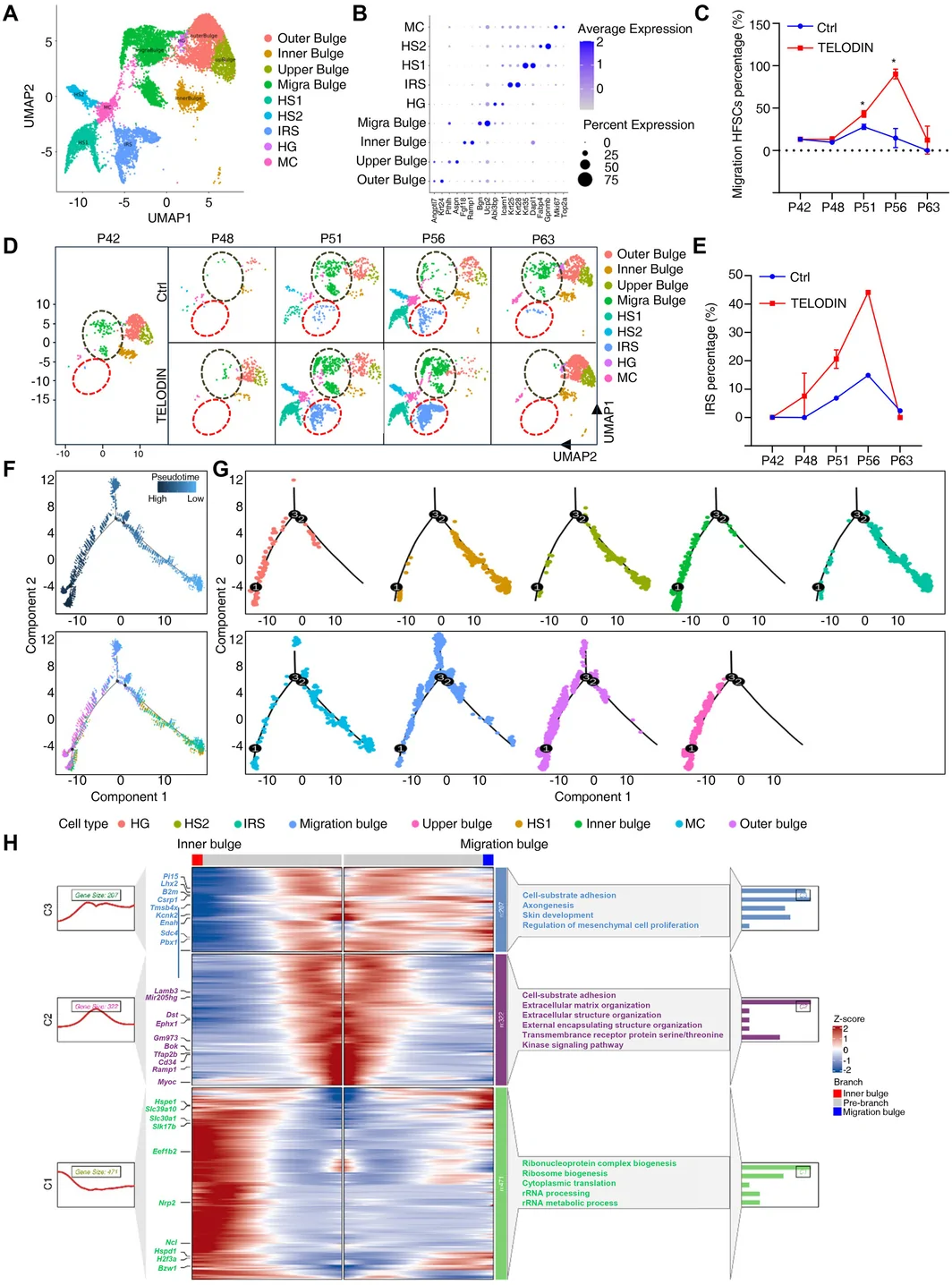
Single‐cell transcriptomic changes in bHFSCs subpopulation treated with TELODIN. (A) UMAP plot showing the nine cell types in mouse bHFSCs. Cell types were labeled on the right. (B) Dot plots displayed representative gene expression for each bHFSCs cell type. (C) Percentage of migration bulge cell at indicated time points (days). (D) UMAP plot depicted bHFSCs cell composition at indicated time points. Red dashed‐line circle indicates IRS and black dashed‐line circle indicates migration bulge. (E) Line plots compared IRS cell percentages between control and TELODIN‐treated mice over time. (F) Pseudotime trajectory analysis of cells in HFs, the inferred pseudotime for each cell type was shown in upper panel and the cell types along the pseudotime trajectories were shown in the lower panel. (G) The distribution of HF subclusters along with the pseudotime trajectory was shown. (H) Heatmaps display gene expression profiles and pseudotime trajectories of bHFSCs subclusters (Control vs. TELODIN). Enriched GO terms for each cluster were listed on the right. *p < 0.05.

High‐resolution clustering of germinative layer cells (GCs) and bHFSCs identified nine major bHFSCs subpopulations: outer bulge, upper bulge, inner bulge, migratory bulge, hair germ, inner root sheath (IRS), hair shaft 1 (HS1), hair shaft 2 (HS2), and mitotic cells (MCs) (Figure 4A,B). TELODIN‐induced bulge cell differentiation and migration from P48 to P56 (Figure 4C,D), concomitant with hair germ cell proliferation. TELODIN significantly expanded IRS cells, which are derived from proliferating GCs (Figure 4D,E). IRS cells exhibited more differentially expressed genes (DEGs) (Figure S11A), particularly those related to cell cycle, cell proliferation, extracellular matrix organization, and cell adhesion (Figure S11B). Inferred pseudotime for each cell type is shown in the top panel, and the corresponding cell types along the pseudotime trajectories are shown in the bottom panel (Figure 4F). The distribution of bHFSCs subpopulations along the pseudotime is shown in Figure 4G. Additionally, the distribution of bHFSCs subclusters along the pseudotime trajectory revealed that the migratory bulge population was markedly increased upon TELODIN treatment (Figure S11C). Cell type‐specific gene expression profiles along the differentiation trajectory identified three distinct gene clusters: Cluster 1 contains genes associated with ribonucleoprotein complex biogenesis and cytoplasmic translation; Cluster 2 contains genes associated with extracellular matrix organization and kinase signaling pathways; Cluster 3 contains genes associated with skin development, cell‐substrate adhesion, and regulation of mesenchymal proliferation (Figure 4H). These findings demonstrate the multifaceted roles of TELODIN in bHFSCs mobilization, hair cycle acceleration, and bHFSCs differentiation.

## 3 Discussion

Although telomerase deficiency causes HF aging (Sarin et al. 2005), it remains poorly understood whether telomere maintenance can promote hair growth. In this study, we report that TPP1 overexpression recruits telomerase and thereby accelerates hair growth in mice. This is achieved by accelerating the hair follicle transition from quiescent telogen to proliferative anagen through telomere maintenance. Remarkably, topical application of TELODIN, a synthetic octapeptide that inhibits TPP1 degradation and increases telomere capping, similarly accelerates hair regeneration. Using single‐cell transcriptomics and immunofluorescence analysis, we demonstrated that TELODIN not only accelerates the hair cycle but also promotes the differentiation and migratory potential of bHFSCs. These findings identify telomere capping and elongation as key regulators of bHFSCs activation and anagen entry during hair regeneration.

### 3.1 Increasing TPP1 Drives the Hair Growth Cycle by Activating bHFSCs

In humans, stem cell telomerase activity and telomere length decline during HF aging (Stone et al. 2021). Telomerase deficiency underpins hair loss, graying, and compromised regeneration in mice (Buckingham and Klingelhutz 2011; Flores et al. 2005; Liu et al. 2019; Siegl‐Cachedenier et al. 2007). Overexpression of telomerase TERT in mouse skin epithelium activates bulge stem cells and promotes hair growth (Jan et al. 2012; Sarin et al. 2005; Siegl‐Cachedenier et al. 2007). Intriguingly, TPP1 deficiency impairs HF morphogenesis (Tejera et al. 2010), and splicing mutations are associated with caudal dysgenesis (Vlangos et al. 2009). Our prior work revealed that TPP1 poly‐ubiquitination triggers telomere uncapping and DNA damage, leading to cellular senescence and pulmonary fibrosis (Wang et al. 2020), consistent with RNF8‐mediated TPP1 stabilization maintaining telomere integrity (Rai et al. 2011). Our present work shows that *Acd* overexpression accelerates HF growth, HF reentry into the hair cycle, and bHFSCs proliferation by reducing telomeric DNA damage (Figure 1 and Figure S2), identifying TPP1 as a novel critical regulator of hair regeneration. Importantly, evidence for TPP1 deficiency derives mainly from murine models, not human cohorts. By addressing a therapeutic strategy to counter telomere erosion‐mediated aging, we propose TPP1‐based intervention for age‐related androgenetic alopecia and other non‐scarring alopecias through stabilizing telomeres in quiescent stem cells. These findings establish telomere stability as a crucial determinant of bHFSCs activation and anagen entry during hair growth.

### 3.2 Specific Inhibition of TPP1 Degradation by TELODIN Promotes bHFSCs Proliferation, Differentiation, and Migration

Reentry into the HF cycle triggers hair regeneration (Ji et al. 2021), as a new HF cycle begins with the telogen‐to‐anagen transition (Alonso and Fuchs 2006). In mice, the first telogen is brief (1–2 days, occurring between P19 and P21 on the mid back), whereas the second telogen lasts over 2 weeks (starting at P42) (Alonso and Fuchs 2006). To assess TELODIN's effect on hair regeneration, we shaved the dorsal skin of mice at P42 and topically applied TELODIN. We found that both TPP1 expression and stabilization accelerate the telogen‐to‐anagen transition and promote hair regeneration (Figures 1 and 2). Supporting this, scRNA‐seq confirmed that TELODIN‐treated mice exhibit increased anagen cells and reduced telogen cells at P48 and P56 (Figure 2), consistent with a recently reported telomere‐homologous oligonucleotide (T‐oligo) in enhancing melanogenesis and skin pigmentation (Hadshiew et al. 2008). As HFSCs reside in the bulge and hair germ (Fuchs 2007), quiescent bHFSCs and primed hair germ cells coordinate to drive HF cycling (Cotsarelis et al. 1990; Deschene et al. 2014; Fuchs 2009; Watt and Jensen 2009) and HFSCs activation and microenvironmental cues collectively regulate HF bulb regeneration across cycling phases (Alonso and Fuchs 2006). Telomere maintenance via TPP1 stabilization using TELODIN may provide the mechanism for bHFSCs activation, as evidenced by elevated Pcna expression and an expanded bHFSCs pool (Figures 3 and 4). Moreover, scRNA‐seq documented that TELODIN‐responsive bHFSCs subpopulations upregulated gene signatures associated with cell motility (e.g., Rhob, Rac1, Akt1) (Figure 4C and Figure S10D), illustrating enhanced migratory potential without ruling out functional migration of activated HFSCs. These data indicate that TELODIN attenuates telomeric DNA damage and activates bHFSCs to promote hair growth.

### 3.3 Telomere‐Triggered Signaling Pathways Mediating HFSCs Proliferation and Differentiation During Telogen‐to‐Anagen Transition

HFSCs are long‐lived proliferative cells that accumulate cellular damage and epigenetic alterations during repetitive HF cycling, ultimately leading to aging‐related quiescence (Matsumura et al. 2016). Without sufficient and functional HFSCs, HF cycling and regeneration become disrupted, causing progressive HF loss and permanent alopecia (Harries et al. 2018). Our findings reveal that TELODIN increased TPP1 expression, suggesting that TELODIN‐induced TPP1 promotes TERT activity to drive bHFSCs proliferative potential and HF cycling. The essential role of TERT was demonstrated by the failure of TELODIN to rescue hair regeneration in *Tert* knockout mice (Figures S7 and S8). These results indicate that functional TERT is critical for TELODIN‐mediated bHFSCs activation. Consistent with previous findings that spontaneous 53BP1 foci accumulate in bHFSCs and epidermal cells during aging (Schuler and Rube 2013), we found that telomeric DNA damage was significantly reduced by TELODIN treatment (Figure 3).

## 4 Limitations and Conclusions

It is noteworthy that our studies used young mice (P42) with minimal telomere damage in HFSCs, raising the question of whether TPP1‐mediated TERT activation operates via canonical telomere elongation or noncanonical functions. Given that TERT overexpression promotes hair growth independently of TERC (Sarin et al. 2005) and regulates signal transduction, gene transcription, and oxidative defense (Rosen et al. 2020; Segal‐Bendirdjian and Geli 2019), further studies are required to investigate TPP1‐induced TERC dependent versus independent signaling. In addition, as TPP1 overexpression alters the checkpoint thresholds of cellular responses, thereby bypassing checkpoint activation and allowing cell division to proceed (Hong et al. 2022), it is critical to employ inducible, regulated TPP1 expression in future investigations. Furthermore, previous studies, including ours, have shown that telomere shortening serves as a checkpoint for epithelial stem cell division, preventing short telomeres from being transmitted to daughter cells (Choi et al. 2020; Nabhan et al. 2018; Wang et al. 2020). Future research is needed to clarify the possibility that TPP1 directly relieves telomere‐mediated restraint within bHFSCs or indirectly stimulates their activation through intercellular interactions.

In conclusion, we demonstrate that both conditional induction of TPP1 in keratinocytes and topical application of TELODIN facilitate bHFSCs proliferation, differentiation, and migratory potential, accelerating telogen‐to‐anagen transition and hair regeneration. These findings provide a paradigm for TELODIN‐based TPP1 stabilization and telomere maintenance to counteract hair loss stemming from hair follicle stem cell replicative senescence.

## 5 Materials and Methods

### 5.1 Animals and Treatment

*Acd* ^*flox/flox*^ mice were generated by Cyagen Biosciences (Suzhou, China). *Keratin 14‐Cre* (*Krt14‐Cre*) mice were obtained from GemPharmatech Co. Ltd. (Nanjing, China). *Tert* knockout mice (C57BL/6J background) (Erdmann et al. 2004) and wild type C57BL/6 mice were maintained under specific pathogen‐free (SPF) conditions at the Animal Centre of Hangzhou Normal University (Hangzhou, China). All animal procedures were approved by the Institutional Animal Care and Ethics Committee at Hangzhou Normal University (Approval number: HSD20200101). For the hair regeneration assays, dorsal hair was depilated at P42 using a 3:1 rosin‐paraffin mixture. TELODIN peptide and control compounds were synthesized by KE Biochem (Shanghai, China). Alpha ketoglutarate (α‐KG) was purchased from MilliporeSigma (Burlington, MA, USA). Test solutions were prepared by dissolving control (1 mg), TELODIN (1 mg), or α‐KG (46.7 mg) in 1 mL ddH_2_O, followed by mixing with 9 mL vehicle solution (containing 700 mL propylene glycol, 3.5 g sodium laurate, 5.0 g lauryl azone, 2.0 g sodium methylate, and 0.5 g sorbic acid per liter ddH_2_O). Treatments were topically applied to shaved dorsal skin.

### 5.2 Hair Cycle Analysis

Hair regrowth was quantified on a 0%–100% scale by assessing both skin pigmentation and hair shaft density, as previously described (Li et al. 2020). The scoring criteria were as follows:

0%: No visible hair growth or pigmentation.

50%: Approximately half of the dorsal skin area covered with hair/pigmentation.

70%: Significant (> 2/3) but incomplete coverage.

100%: Full hair coat recovery with complete pigmentation.

### 5.3 Hematoxylin and Eosin (H&E) Staining, Masson Staining, and Immunofluorescence (IF) Staining Assay

Dorsal skin samples were fixed in 4% paraformaldehyde (PFA). H&E staining, Masson staining, and IF staining were performed as previously described (Wang et al. 2020). For IF staining, the following primary antibodies were used: anti‐Ki67 (Cat# ab15580, 1:100 dilution; Abcam); anti‐Lgr6 (Cat# sc‐393010, 1:50 dilution; Santa Cruz); anti‐Integrin α6 (Cat# 14‐0495‐82, 1:100 dilution; ThermoFisher); anti‐CD34 (Cat# 07‐3403, 1:100 dilution; ThermoFisher); anti‐S100a6 (Cat# MA5‐32511, 1:100 dilution; ThermoFisher); and anti‐53BP1 (Cat# ab36823, 1:100 dilution; Abcam). The corresponding fluorescence secondary antibodies were: anti‐mouse IgG (H + L) and rabbit IgG (H + L), Alexa Fluor 488 (Cat# A28175, 1:500 dilution; ThermoFisher), Alexa Fluor 555 (Cat# A28180, 1:500 dilution; ThermoFisher), Alexa Fluor 488 (Cat# A27034, 1:500 dilution; ThermoFisher), and Alexa Fluor 555 (Cat# A27039, 1:500 dilution; ThermoFisher).

### 5.4 Telomere DNA Damage Assay

Skin tissue samples were fixed, paraffin‐embedded, and sectioned. Sections mounted on coverslips were blocked for 30 min with blocking solution (1 mg/mL BSA, 3% goat serum, 0.1% Triton X 100, 1 mM ethylenediaminetetraacetic acid [EDTA] [pH 8.0]). Primary antibody incubation was performed using anti‐53BP1 antibody (Cat# ab36923; Abcam) for 1 h at room temperature, followed by PBS washes. Coverslips were then incubated with Alexa Fluor 488‐conjugated secondary antibody for 40 min at room temperature and washed with PBS. For telomere detection, sections were postfixed with 2% PFA for 5 min. The hybridization mixture containing the 5′ Cy3‐OO‐(CCCTTA)_3_‐peptide nucleic acid (PNA) probe (70% [v/v] formamide, 0.5% blocking reagent, 10 mM Tris–HCl [pH 7.2]) was applied. Denaturation was conducted at 80°C for 3–10 min, followed by overnight hybridization at 4°C in the dark. Post‐hybridization washes included two washes with 70% (v/v) formamide/10 mM Tris–HCl pH 7.2, followed by three PBS washes. Nuclear counterstaining was performed with DAPI (4′, 6‐diamidino‐2‐phenylindole).

### 5.5 Quantitative‐Fluorescence In Situ Hybridization (Q‐FISH)

Telomeres were determined by Q‐FISH according to the protocol essentially as described previously (Wang et al. 2020). Briefly, in paraffin embedding and sectioning of the dorsal skin tissues, the sections were fixed, dehydrated, and hybridized with Cy3‐OO‐(CCCTTA)_3_‐peptide nucleic acid (PNA) probe (Panagene) and DAPI, which was followed by fluorescence imaging and quantification.

### 5.6 Isolation of HFSCs by Fluorescence‐Activated Cell Sorting (FACS)

Following anesthesia, dorsal skin was excised and subcutaneous fat was carefully removed using surgical scissors. Tissues were minced into ~1 mm^2^ fragments and digested in 5 mL Hank's balanced salt solution (HBSS) containing 0.25% collagenase IV (w/v) at 37°C for 2.5 h with constant agitation (150 rpm). The digestion was terminated by adding 0.05% trypsin (final concentration) for 8 min at 37°C. The cell suspension was sequentially filtered through 70 and 40 μm cell strainers, and centrifuged at 700 *g* for 13 min at 4°C. Pelleted cells were resuspended in 0.5–1 mL Dulbecco's PBS (D‐PBS) and stained in a 1.5 mL Eppendorf (EP) tube with the following fluorochrome‐conjugated antibodies: phycoerythrin (PE)‐conjugated anti‐Cd34 antibody (Cat# 128610; BioLegend Inc., San Diego, CA, USA), fluorescein isothiocyanate (FITC)‐conjugated anti‐Itga6 antibody (Cat# 11‐0495‐82; eBioscience Inc., San Diego, CA, USA), peridinin‐chlorophyll protein (PerCP)‐conjugated anti‐Sca‐1 antibody (Cat# 45‐5981‐82; Invitrogen, Carlsbad, CA, USA), and Cy7‐conjugated anti‐Itgb1 (Cat# ab95622; Abcam).

### 5.7 RNA Extraction and Quantitative Real‐Time Polymerase Chain Reaction (qPCR)

Total RNA was extracted using TRIzol reagent (Cat# CW0580; CWBio, Beijing, China) and reverse transcribed using the HiFiScript cDNA Synthesis Kit (Cat# CW2659; CWBio). Quantitative PCR was performed using the UltraSYBR Mixture with Low Rox (Cat# CW2601; CWBio). The primer sequences used were listed in Table S1.

### 5.8 Protein Extraction and Immunoblotting

Skin tissue samples (1 cm^2^) were homogenized in radioimmunoprecipitation assay (RIPA) lysis buffer (Beyotime Biotechnology Co. Ltd., Shanghai, China) using a tissue homogenizer. Protein concentrations were determined using a bicinchoninic acid (BCA) assay kit (Beyotime Biotechnology Co. Ltd.). Equal amounts of protein (50 μg) were separated by 8%–10% sodium dodecyl sulfate‐polyacrylamide gel electrophoresis (SDS‐PAGE) and transferred to PVDF membranes. The following primary antibodies were used for immunoblotting: Anti‐E‐cadherin (Cat# 20874‐1‐AP; ProteinTech Group Inc., Rosemont, IL, USA), anti‐TPP1 (Cat# NBP2‐45475; Novus Biologicals LLC, Centennial, CO, USA), anti‐Pcna (Cat# sc‐56; Santa Cruz Biotechnology, Santa Cruz, CA, USA), and anti‐Gapdh (Cat# G9545; MilliporeSigma) antibodies.

### 5.9 Tissue Dissociation and Preparation of Single‐Cell Suspensions

Fresh skin tissues (1 mm^2^) were collected from P42 to P63 mice, transferred to a Petri dish on wet ice containing 1 × PBS (without RNase and Ca, Mg ions), and washed to remove blood and debris. Tissues were minced into 0.5 mm^2^ pieces, digested in 0.35% collagenase IV, 2 mg/mL papain, and 120 Units/mL DNase I at 37°C for 20 min (100 rpm), and quenched with PBS containing 10% fetal bovine serum. The suspension was gently pipetted 5–10 times, filtered through a 70 μm then 30 μm sieve and centrifuged at 300 × *g* for 5 min at 4°C. Erythrocytes were lysed using 1× RBC lysis buffer (MACS) for 2–10 min on ice, followed by dead cell removal with MicroBeads (MACS) and MS Columns. Viability (> 85%) and concentration (700–1200 cells/μL) were confirmed by trypan blue staining and Countess II counting.

### 5.10 Chromium 10× Genomics Library and Sequencing

Single‐cell suspensions were loaded onto a 10 × Chromium chip (10 × Genomics Chromium Single‐Cell 3′ kit V3), targeting ~8000 cells. cDNA amplification and library preparation followed the manufacturer's protocol. Libraries were sequenced on an Illumina NovaSeq 6000 (by LC‐Bio Technology, Hangzhou, China) with paired‐end 150 bp reads, achieving a median depth of ≥ 20,000 reads per cell.

### 5.11 Bioinformatics Analysis

Raw Illumina sequencing data were demultiplexed and converted to FASTQ format using bcl2fastq (version 5.0.1). scRNA‐seq reads were aligned to the reference genome (GRCh38/mm10) and quantified using CellRanger (v7.2.0; 10 × Genomics). The gene‐cell matrix was imported into Seurat (v4.1.0) for quality control (thresholds: > 500 genes/cell and mitochondrial gene percentage < 25%), normalization, and clustering. Batch effects were corrected with Harmony (v1.0), and dimensionality reduction was performed using UMAP (n_neighbors = 30, min_dist = 0.3). Interactive analysis was conducted via OmicStudio (LC‐BIO, Hangzhou).

### 5.12 Statistical Analyses

All experiments were repeated at least three times. Data are expressed as mean ± SEM (standard error of the mean). Comparisons between groups were analyzed using a two‐tailed Student's *t*‐test, with statistical significance defined as *p < 0.05, **p < 0.01, and ***p < 0.001. Specific statistical tests, sample sizes (*n*), and exact *p* values are provided in figure legends. Analyses were performed using GraphPad Prism (v7.0; GraphPad Software).

## Author Contributions

L.W. and B.Z. carried out gene expression, tissue, cell, and gene analyses. B.Z., J.Y., Y.L., and X.X. did the treatment and tissue process of mice. L.Y., J.X., H.L., Y.Y., L.G., J.Z., T.S., H.X., J.Z., H.W., and J.L. assisted with animal work and preparation of experimental materials. X.H. assisted with helpful discussions. L.W. and J.‐P.L. summarized results, interpreted data, and wrote the paper. J.‐P.L. conceived the study, designed, and supervised the experiments.

## Funding

This work was supported by National Key Research and Development Program of China (2022YFA1103800, 2021ZD0202402), National Natural Science Foundation of China (82130044, 91949207, 92149302, 82271594), and Natural Science Foundation of Zhejiang Province (LZ22H250001).

## Conflicts of Interest

J‐.P.L. is the founder of Hangzhou Duanli Biotechnology Company Limited, Hangzhou, Zhejiang, China.

## Supporting information


**Figure S1:** Construction strategy of *Acd*‐overexpressing mice. (A) Outline of the *m* *Acd* ^*flox/flox*^ mice construction strategy. (B) PCR confirms genotypes of WT, heterozygous, and homozygous mice. (C) qPCR analysis of Shelterin complex components expression in skin tissues, *n* = 4, **p < 0.01. (D) Generation strategy for *m* *Acd* ^*flox/flox;Krt14‐Cre*^ mice. *m* *Acd* ^*flox/flox*^ mice are crossed with *Krt14*‐Cre mice to produce homozygous and control offspring.
**Figure S2:** TPP1 activates bHFSCs and prevents telomere DNA damage in bHFSCs. (A) Hair regrowth assay was performed on WT control and *Acd*‐overexpressed mice. The dorsal skin was shaved at P42 (upper panel, female mice; lower panel, male mice) with images captured at indicated time points (P42–P63). (B) Quantitative analysis of the dermis thickness at P42, 48, P51, P58, and P63. (C) Surface expression of Lgr6 and Integrin α6 on sorted bHFSCs was verified by IF staining. Scale bar = 10 μm. (D–D′) qPCR analysis of stem cell markers (Cd34, Foxc1, Itga6, Lgr5, Lgr6, Lhx2, Nfatc1, Sox9, Tcf3, Tcf4, Krt15, and S100a6) was performed on total skin cells (D) and sorted bHFSCs (D′) from WT control (Ctrl) and *Acd* overexpressed mice. (E–E′) Temporal expression patterns of Tpp1 (E) and Integrin α6 (E′) in bHFSCs were analyzed by qPCR at indicated time points (P42–P65). (F–H) Telomere‐associated DNA damage in HFs (F) and sorted bHFSCs (G, H) was assessed by IF‐FISH using 53BP1 antibody (Green) and telomere‐specific probe (Red). Scale bar = 5 μm. All data represent mean ± SEM. *p < 0.05; **p < 0.01; ***p < 0.001.
**Figure S3:** TELODIN promotes telogen‐to‐anagen transition in murine HFs. (A) At P42, the back skin of mice was shaved, and mixtures (control, TELODIN, or α‐KG) were applied daily. Representative images are shown at P42, P49, P53, P59, and P63. (B) HF morphology was analyzed by H&E staining at P63, *n* = 3–4, *p < 0.05; ***p < 0.001. Scale bar = 200 μm. (C) Representative images of positive Ki67 staining at P63 were shown. Scale bar = 25 μm. (D) At P65, the same treatment protocol was repeated, with images taken at P65, P69, P73, P77, and P81. (E) HF morphology was analyzed by H&E staining at P77, *n* = 5–8, *p < 0.05; ***p < 0.001. Scale bar = 200 μm. (F) Representative images of positive Ki67 staining at P77 were shown, *n* = 9–12, ***p < 0.001. Scale bar = 25 μm.
**Figure S4:** TELODIN accelerates hair regeneration and the hair cycle in female mice. (A) The back skin of mice in control and TELODIN groups was shaved at P42. A mixture containing vehicle (control) or TELODIN was applied daily. Representative images at P42–P59 are shown (right panel: hair coverage percentage, **p < 0.01). (B) H&E stained back skin sections at P42–P63. Yellow dotted arrows indicated HFs length and black arrows indicated dermal thickness. Scale bar = 100 μm. (C–E) Quantification of HF number (C), dermal thickness (D), and HF length (E) was summarized from five mice, *p < 0.05; **p < 0.01.
**Figure S5:** Enhanced bHFSCs promotes cyclic hair regeneration and reduced telomere DNA damage. (A) bHFSCs were isolated from female mouse back skin by flow cytometry at P42, P46, P52, P58, and P64 using the following markers: Itga6^high^, Itgb1^+^, Sca‐1^−^, and Cd34^+^. (B) The percentage of bHFSCs among total cells was quantified, *n* = 5, **p < 0.01; ***p < 0.001; ns, no significance. (C) Comparative qPCR analysis of stem cell markers (Tcf3, Tcf4, Foxc1, Itga6, Lgr5, S100a6, Sox9, Krt15, Cd34, Lhx2, and Nfatc1) was performed on bulk skin cells and FACS‐purified bHFSCs. (D, E) IF‐FISH analysis with 53BP1 antibody (Green) and telomere probe (Red) assessed telomere DNA damage in paraffin‐embedded skin tissue. Scale bar = 25 μm. (F) Western blot analysis of E‐cadherin (E‐cad), Tpp1, and Pcna expression in skin tissues from control and TELODIN‐treated mice at P47 and P63. Gapdh was used as the loading control. All data represent mean ± SEM. *p < 0.05; **p < 0.01; ***p < 0.001; ns, no significance.
**Figure S6:** The expression levels of markers of bHFSCs are increased by TELODIN treatment. (A–E) bHFSCs were isolated by FACS from mice at P42 (A), P49 (B), P54 (C), P59 (D), and P63 (E) using the following surface markers Sca1^−^, Itga6^high^, Itgb1^high^, and Cd34^+^. The expression of bHFSCs markers (e.g., Lgr5, Sox9) was analyzed by qPCR. *p < 0.05; **p < 0.01; ***p < 0.001.
**Figure S7:** TELODIN does not rescue the defect of hair regeneration caused by *Tert* knockout. (A) Hair regrowth was observed on the dorsal skin of WT and *Tert* knockout mice. The dorsal skin was shaved at P42 with images captured at indicated time points (P42–P60). (B) As compared with control WT animals, TELODIN treatment showed no significant effect on promoting or rescuing the reduced growth of hairs in the *Tert* KO mice of both sexes. *p < 0.05; n.s., no significance.
**Figure S8:** TELODIN administration does not reverse the impaired hair regeneration caused by *Tert* knockout. (A) Histological analysis of dorsal skin collected at P42, P48, P51, P58, and P63 using Masson's trichrome staining. Scale bar = 200 μm. (B) Quantitative analysis of hair follicle number (number/mm), hair follicle length and dermis thickness at P42, 48, P51, P58, and P63. (C) Analysis bHFSCs from mouse back skin performed by flow cytometry at P42, P48, P51, P58, and P63 using the following markers: Itga6^high^, Itgb1^+^, Sca‐1^−^, and Cd34^+^. n.s., no significance.
**Figure S9:** TELODIN does not rescue telomere shortening in *Tert* knockout mice. (A) Representative images of telomere length by q‐FISH. Scale bar = 100 μm. (B) The mean telomere fluorescence intensity per cell per follicle was summarized and displayed. ***p < 0.001; n.s., no significance.
**Figure S10:** Differentially expressed genes between control and TELODIN‐treated mice. (A) Numbers of differentially expressed genes in 13 cell types from control and TELODIN‐treated mice. (B) GO and KEGG analysis of enriched upregulated genes after TELODIN treatment. (C) GSEA revealed significantly upregulated and downregulated signaling pathways in TELODIN‐treated mice compared with control (left panel). The expression patterns of representative pathway‐associated genes are displayed (right panel). (D) Heatmap showed differentially expressed genes related to cell proliferation and migration processes. (E) Gene set enrichment analysis (GSEA) showed cell division, telomere maintenance via telomerase, and telomere maintenance were enriched under TELODIN treatment.
**Figure S11:** Differentially expressed genes of HFSCs between control and TELODIN‐treated mice. (A) Differentially expressed gene counts in eight cell types (control vs. TELODIN). (B) GO and KEGG analysis of enriched upregulated genes after TELODIN treatment. (C) The distribution of HF subclusters along with the pseudotime trajectory was shown.

**Table S1:** Primer sequences.

## Data Availability

The data that support the findings of this study are available from the corresponding author upon reasonable request.
