# Supplementary material for: Shelterin TPP1 Promotes Hair Regeneration Through Activating Bulge Hair Follicle Stem Cells
Source: Aging Cell. 2026 Aug 28;25(9):e70692. doi: 10.1111/acel.70692 (PMC13522991; doi:10.1111/acel.70692)

Figure S1. Construction strategy of *Acd* overexpressing mice.

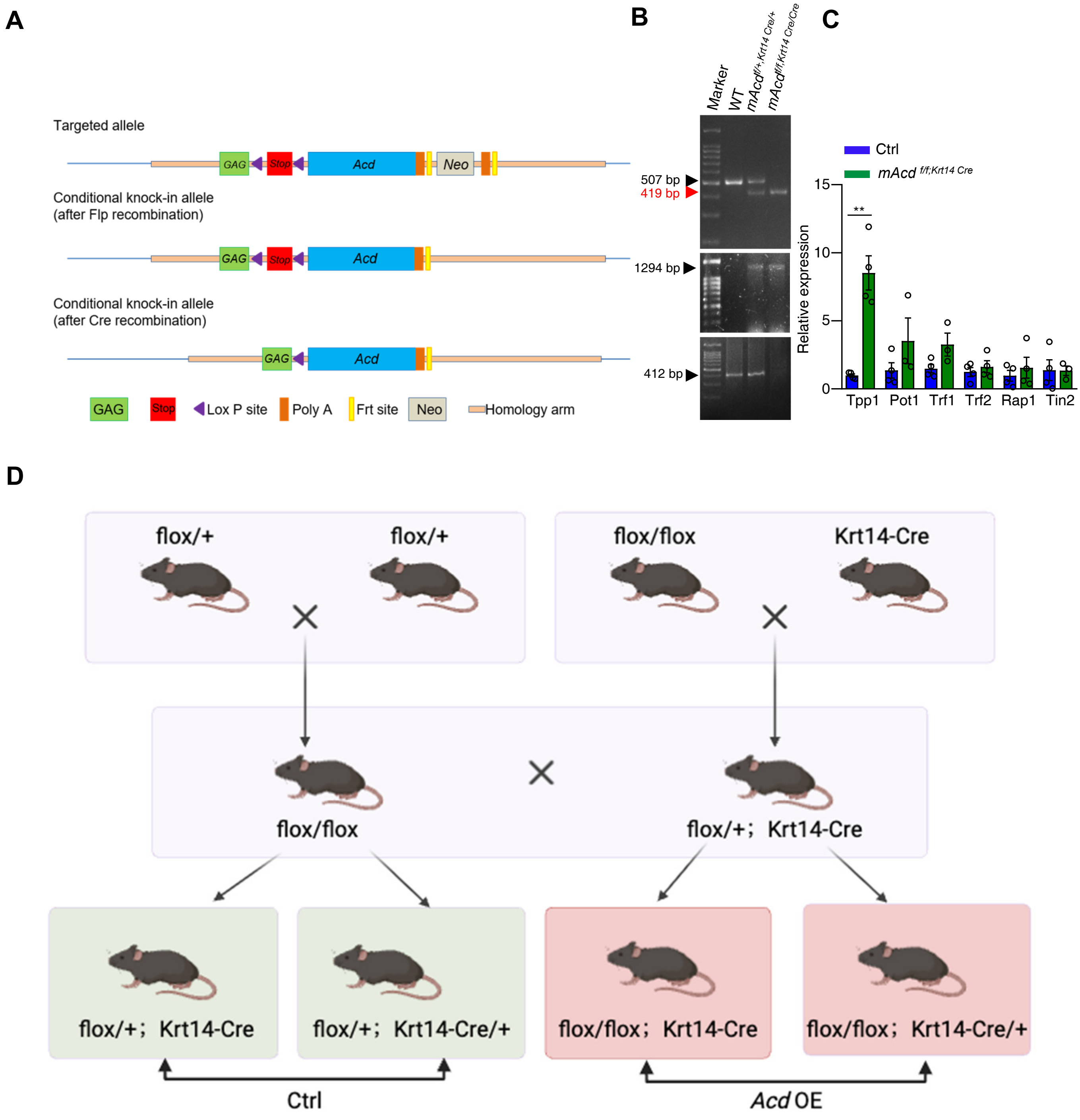

Figure S2. TPP1 activates bHFSCs and prevents telomere DNA damage in bHFSCs.

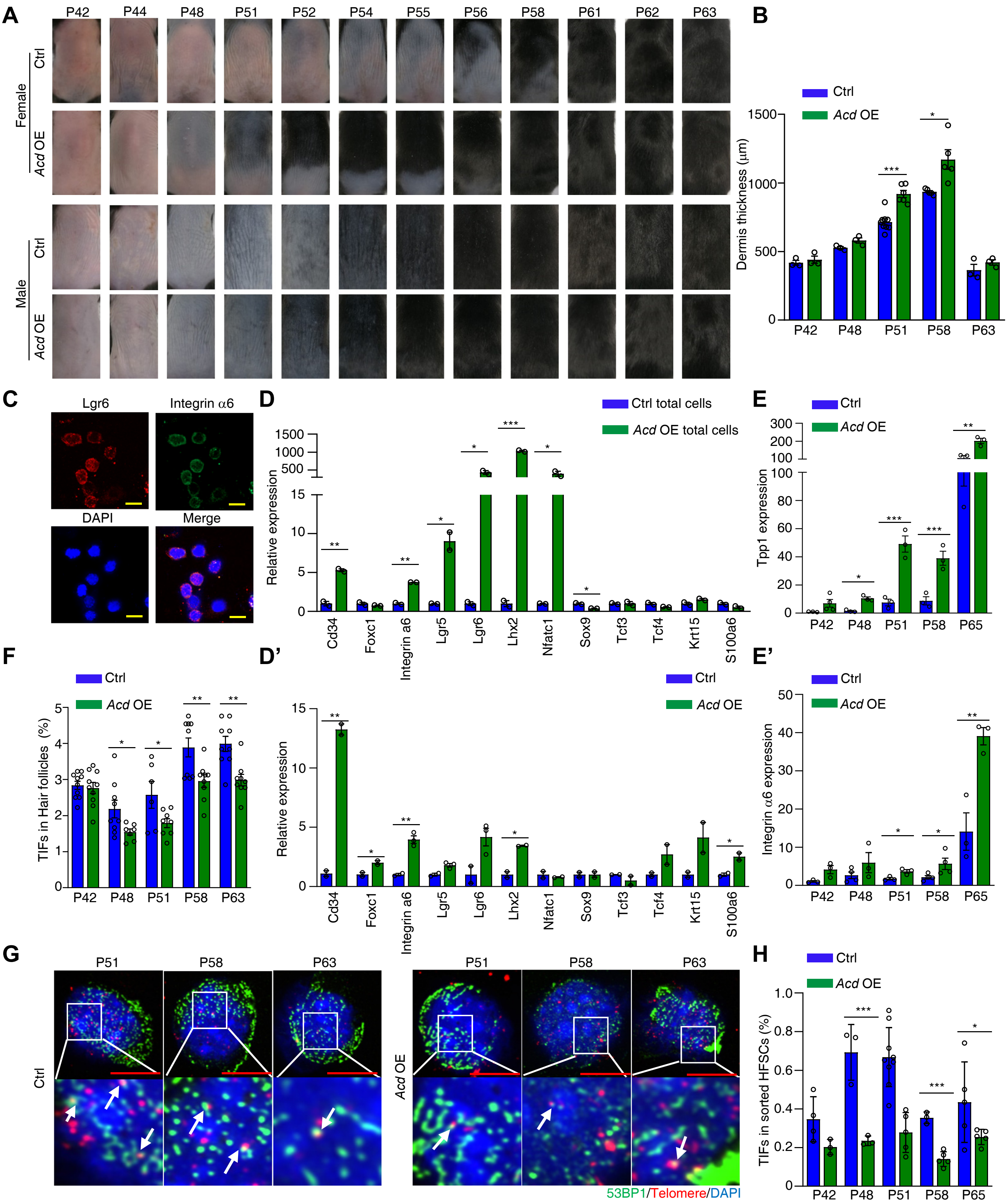

Figure S3. TELODIN promotes telogen-to-anagen transition in murine HFs.

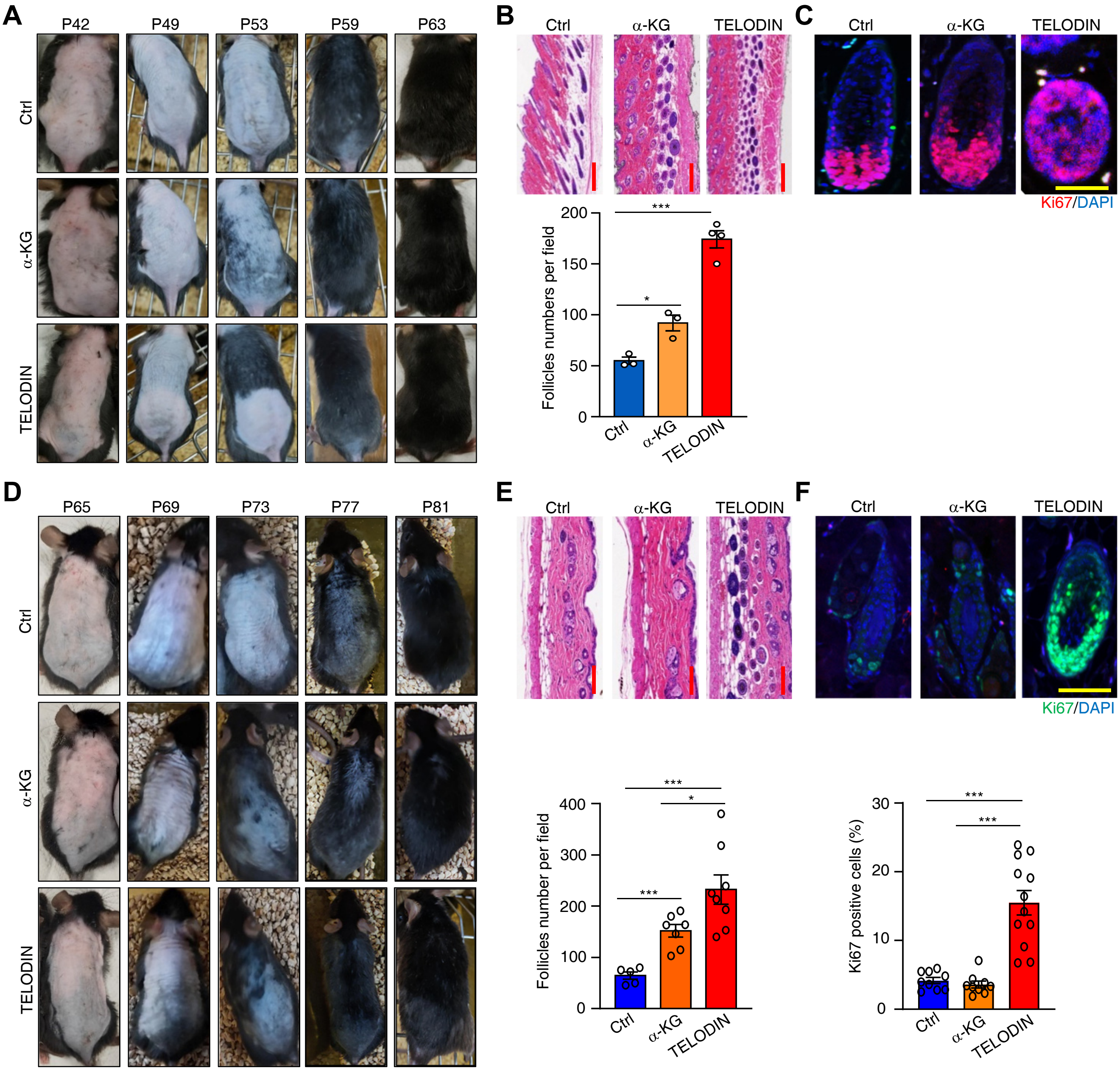

Figure S4. TELODIN accelerates hair regeneration and the hair cycle in female mice.

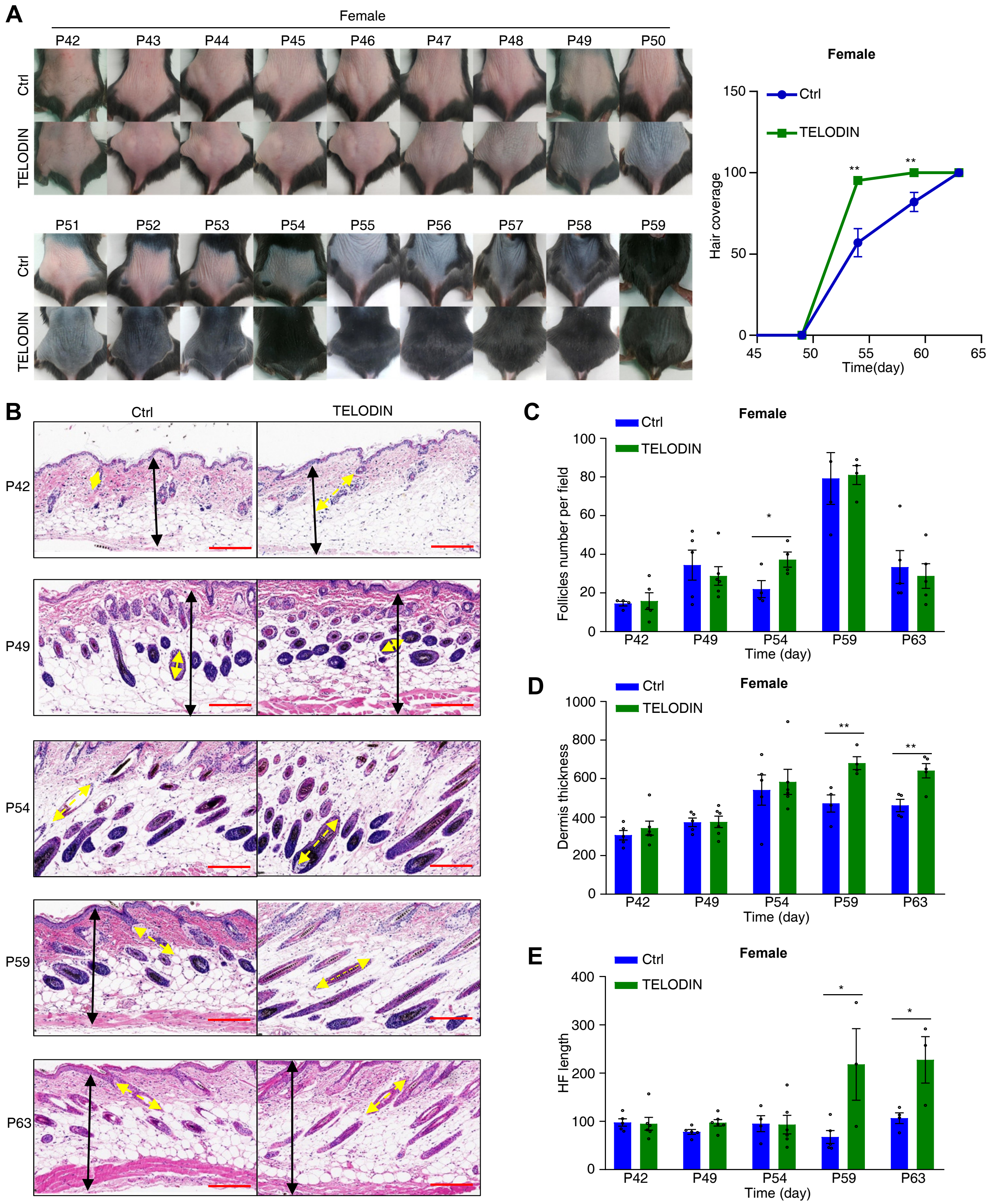

Figure S5. Enhances HFSCs promotes cyclic hair regeneration and reduced telomere DNA damage.

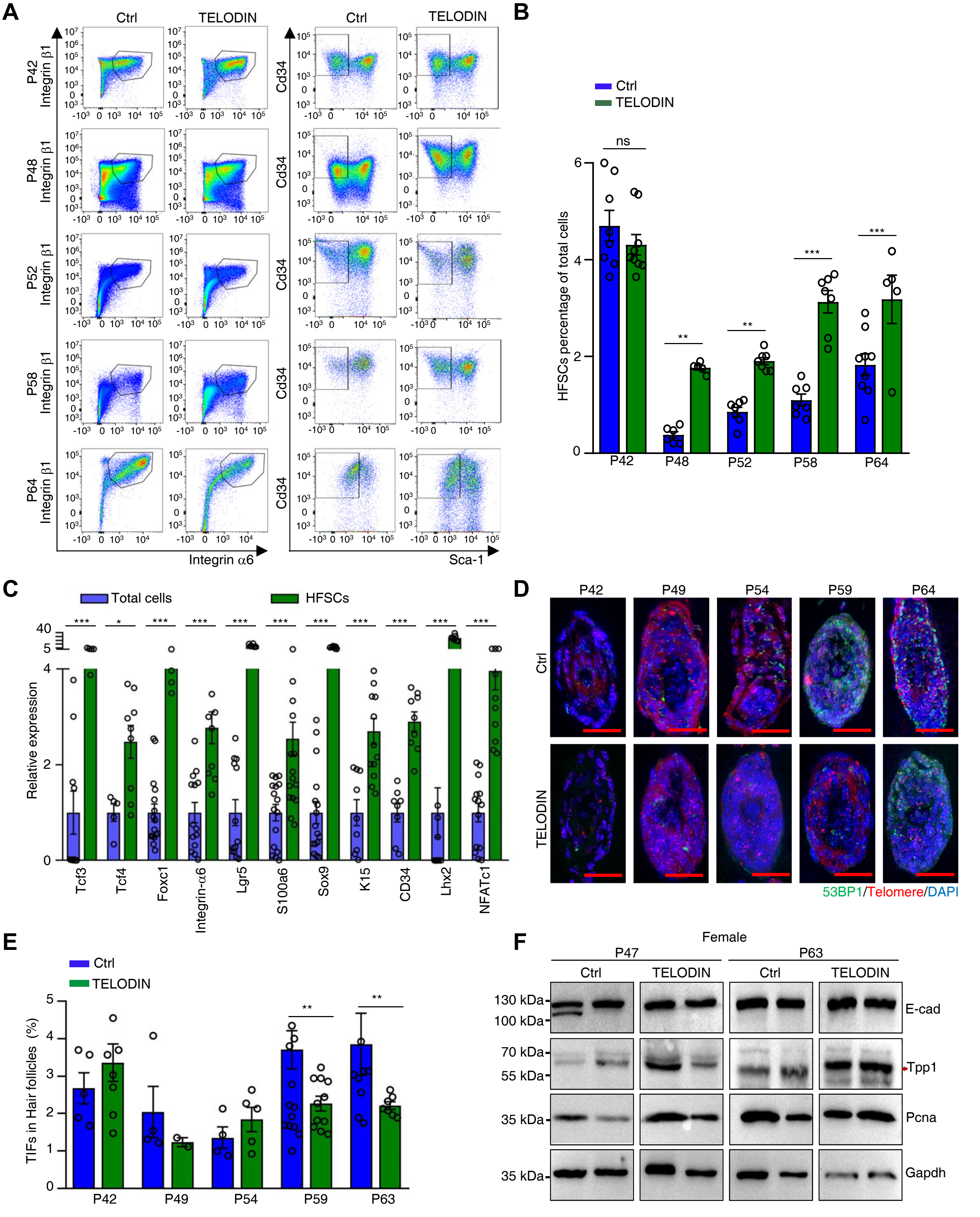

Figure S6. The expression levels of markers of bHFSCs are increased by TELODIN treatment.

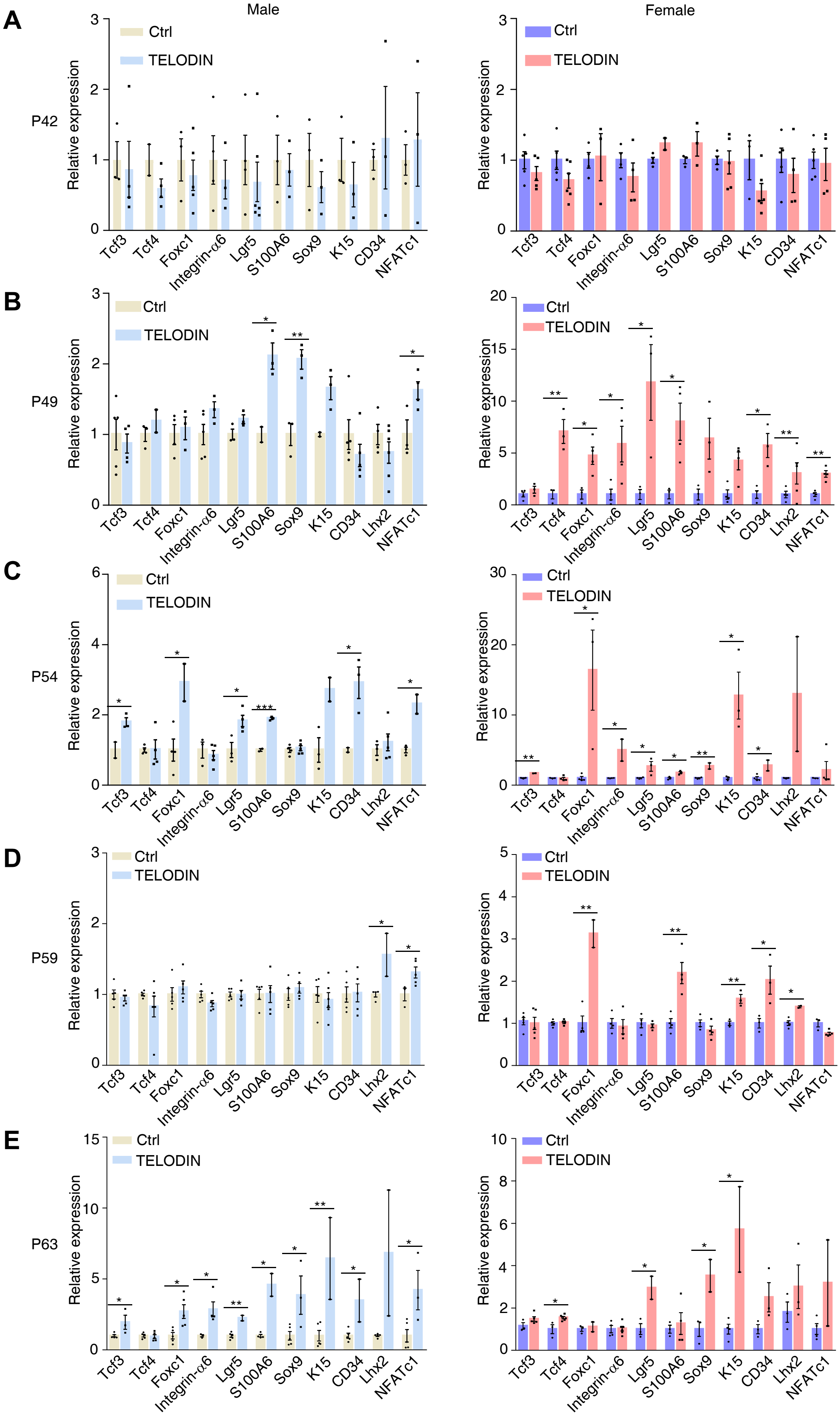

Figure S7. TELODIN does not rescue the defect of hair regeneration caused by *Tert* knockout.

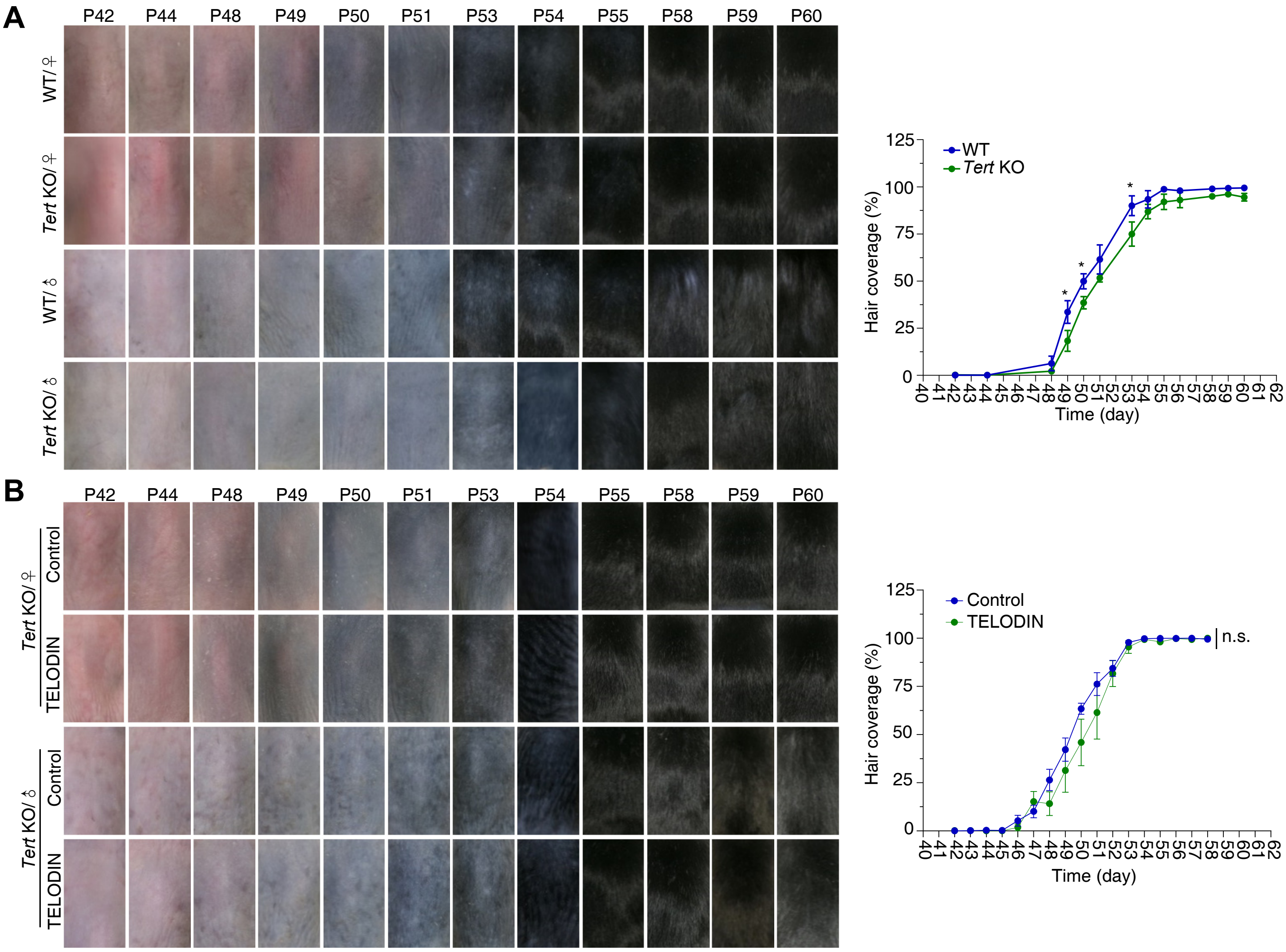

Figure S8. TELODIN administration does not reverse the impaired hair regeneration caused by *Tert* knockout.

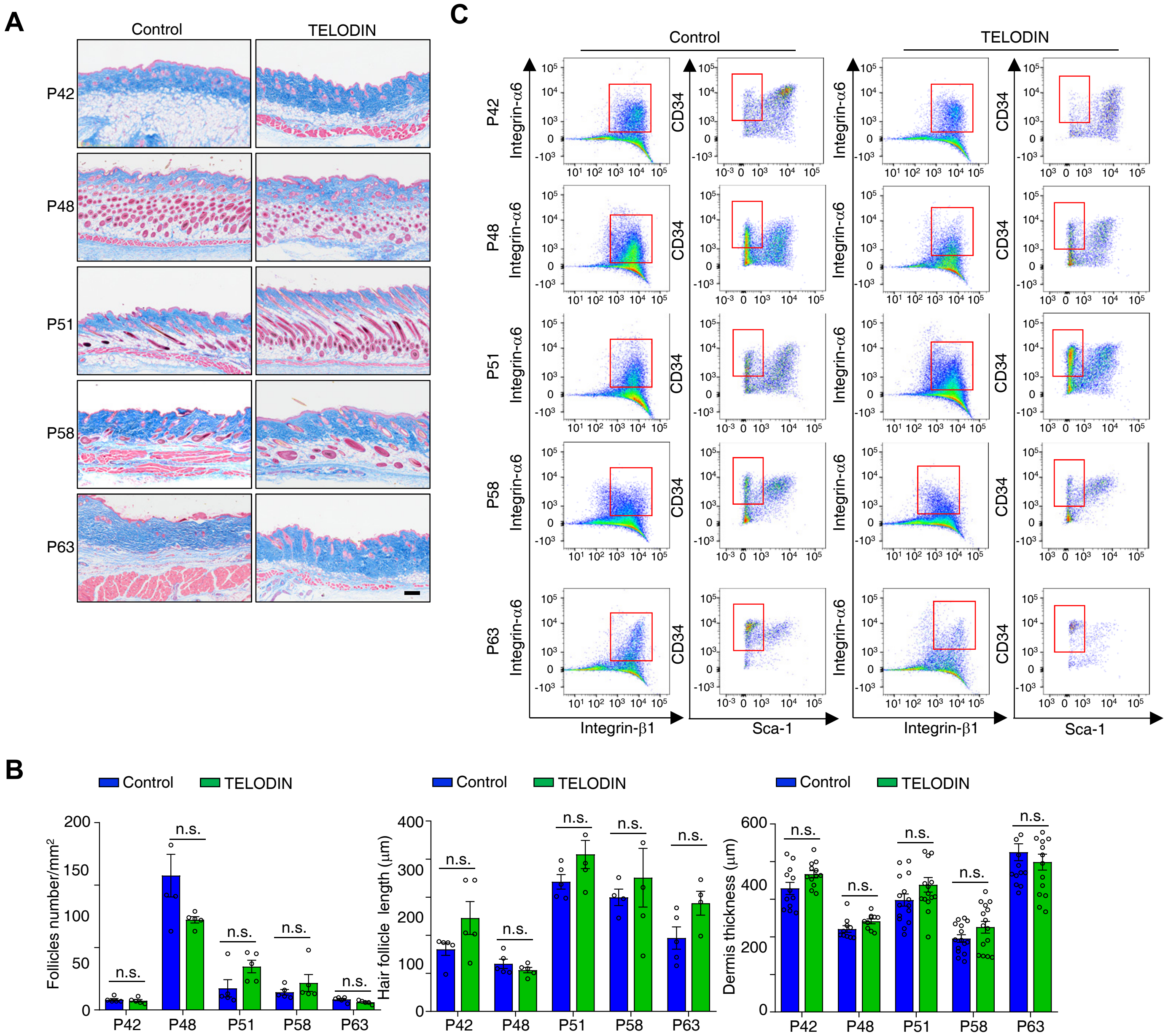

Figure S9. TELODIN does not rescue telomere shortening in *Tert* knockout mice.

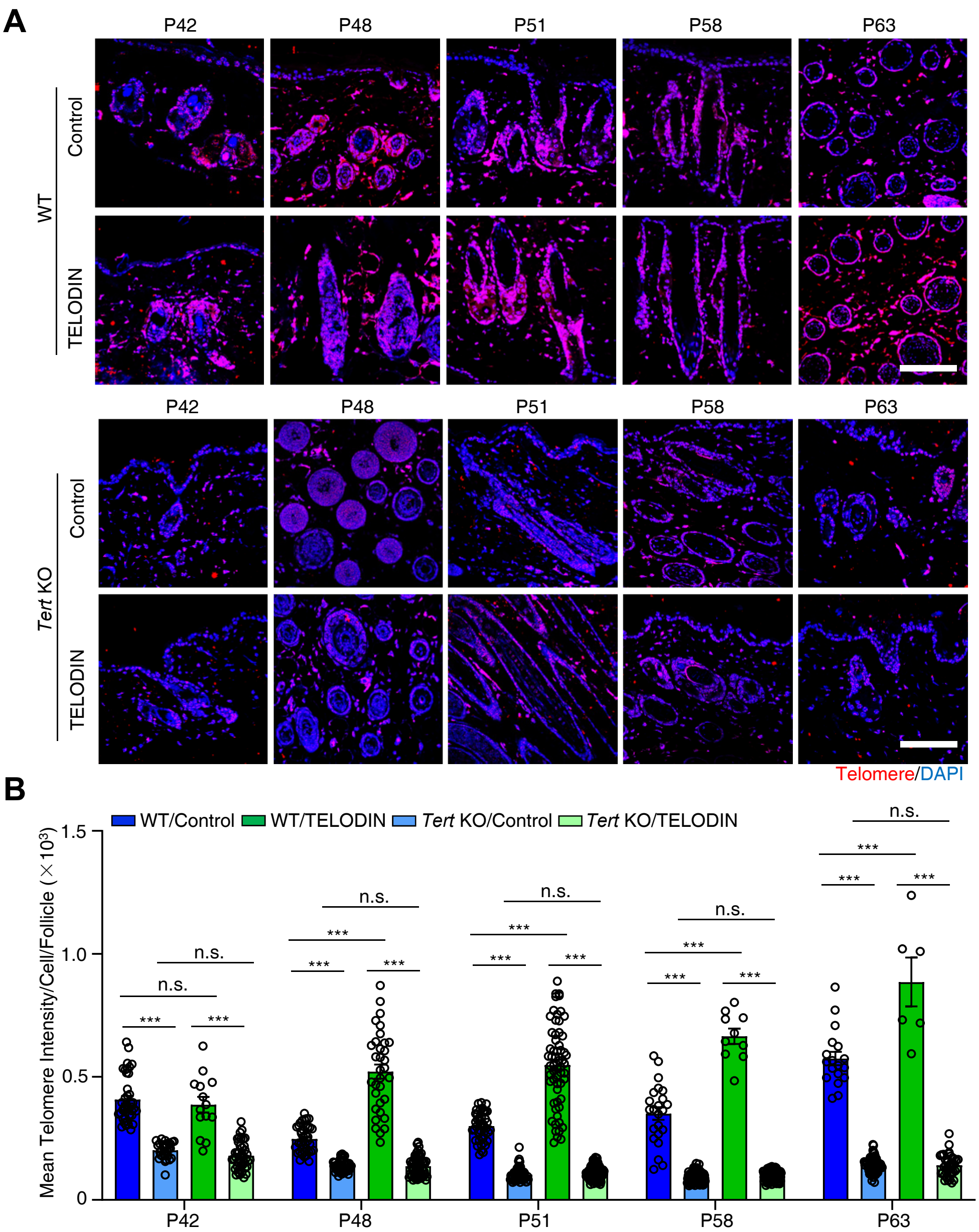

Figure S10. Differentially expressed genes between control and TELODIN-treated mice.

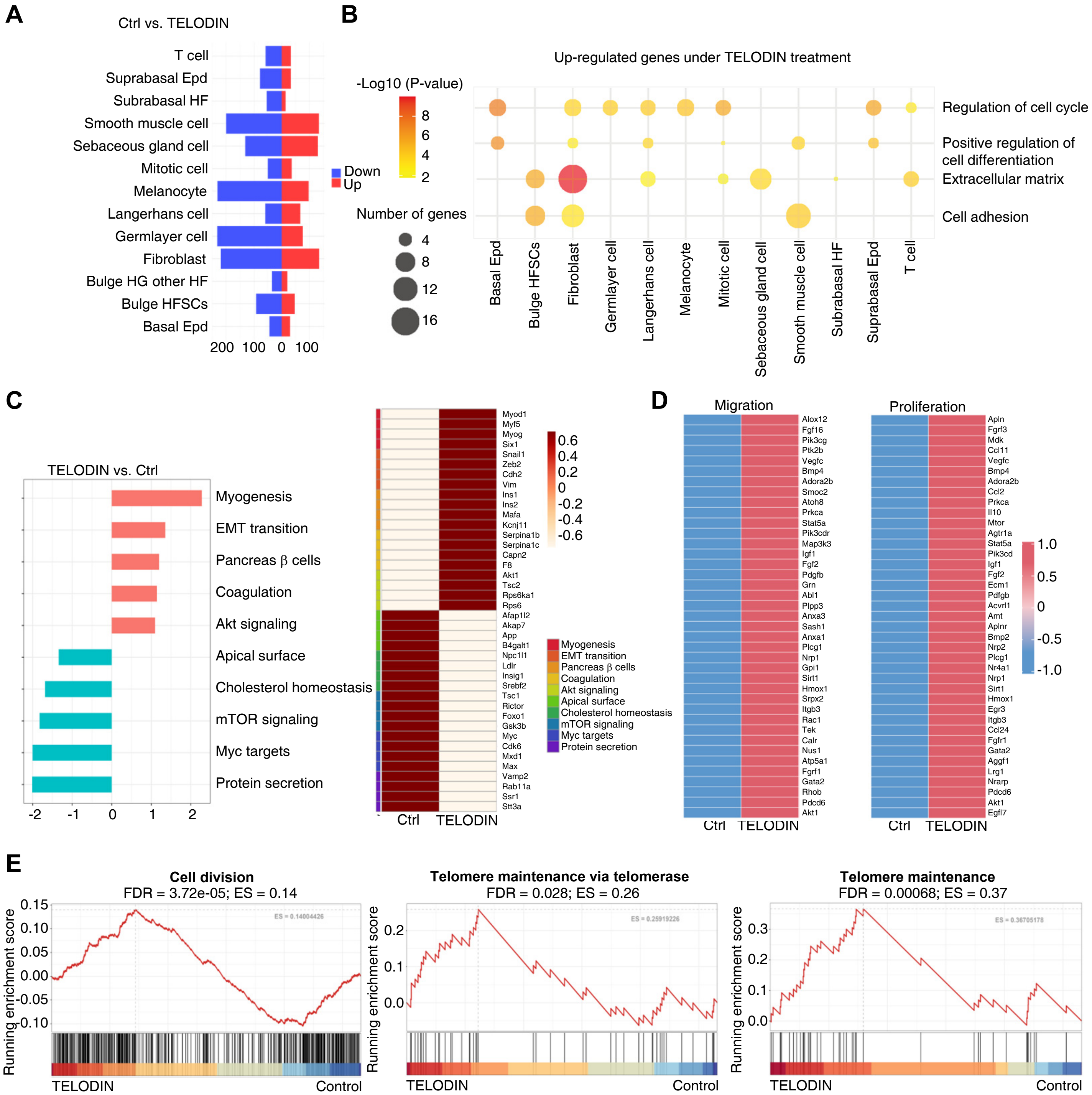

Figure S11. Differentially expressed genes of HFSCs between control and TELODIN-treated mice.

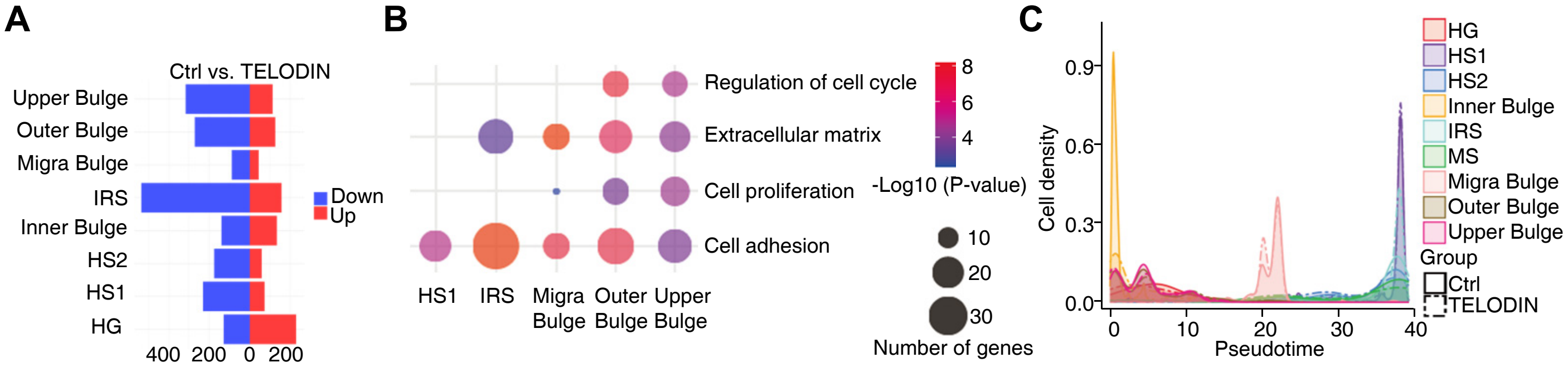

Supplement: Supplementary file 1 [file ACEL-25-e70692-s002.pdf]
