# Supplementary material for: Shelterin TPP1 Promotes Hair Regeneration Through Activating Bulge Hair Follicle Stem Cells
Source: Aging Cell. 2026 Aug 28;25(9):e70692. doi: 10.1111/acel.70692 (PMC13522991; doi:10.1111/acel.70692)
Supplement: Supplementary file 2 [file ACEL-25-e70692-s001.docx]

**Table S1. Primer sequences**

| No. | Name | Sequence |
| --- | --- | --- |
| 1 | Actin-1 | GGCTGTATTCCCCTCCATCG |
| 2 | Actin-2 | CCAGTTGGTAACAATGCCATGT |
| 3 | Trf1-1 | GACAGATTCGGATTCTCCAGAGC |
| 4 | Trf1-2 | AGGCAGAGGAAGTCGAGCA |
| 5 | Trf2-1 | GGTGCTCAAGTTCTATTTCCACG |
| 6 | Trf2-2 | CAAGCAACGCCTGCATGAT |
| 7 | Tpp1-1 | GGGCCGTGTTACTTGTGTCA |
| 8 | Tpp1-2 | CAGCAGGTTAAAGCGATCCAC |
| 9 | Tin2-1 | TTCCCGAAAGTGGTGGAGTTT |
| 10 | Tin2-2 | TGGCCTTTAGGCTCATACACA |
| 11 | Pot1-1 | AGCCTCCGTATGTTAGCAAAGG |
| 12 | Pot1-2 | CCTGTGGAAGCGAACAATGT |
| 13 | Rap1-1 | TGCCTTGTGGAAAGCGATG |
| 14 | Rap1-2 | TGTTCTGTGGCTCTCCGCTAT |
| 15 | S100a6-1 | AAGGAGCTGAAGGAGTTGA |
| 16 | S100a6-2 | CCCAGGAAGGCGACATAC |
| 17 | Cd34-1 | AAGGCTGGGTGAAGACCCTTA |
| 18 | Cd34-2 | TGAATGGCCGTTTCTGGAAGT |
| 19 | Integrin α6-1 | TGCAGAGGGCGAACAGAAC |
| 20 | Integrin α6-2 | GCACACGTCACCACTTTGC |
| 21 | K15-1 | AGCTATTGCAGAGAAAAACCGT |
| 22 | K15-2 | GGTCCGTCTCAGGTCTGTG |
| 23 | Lhx2-1 | CTGTTCCACAGTCTGTCGGG |
| 24 | Lhx2-2 | CAGCAGGTAGTAGCGGTCAG |
| 25 | Lgr5-1 | CCTACTCGAAGACTTACCCAGT |
| 26 | Lgr5-2 | GCATTGGGGTGAATGATAGCA |
| 27 | Sox9-1 | GAGCCGGATCTGAAGAGGGA |
| 28 | Sox9-2 | GCTTGACGTGTGGCTTGTTC |
| 29 | Tcf3-1 | CGCACCAGCAGTACAGATGAG |
| 30 | Tcf3-2 | CAGCTTGGTCTGCGCCTTA |
| 31 | Tcf4-1 | CGAAAAGTTCCTCCGGGTTTG |
| 32 | Tcf4-2 | CGTAGCCGGGCTGATTCAT |
| 33 | Nfatc1-1 | GACCCGGAGTTCGACTTCG |
| 34 | Nfatc1-2 | TGACACTAGGGGACACATAACTG |
| 35 | Foxc1-1 | CCCCGGACAAGAAGATCACTC |
| 36 | Foxc1-2 | AGGTTGTGCCGTATGCTGTTC |
| 37 | Ki67-1 | ATCATTGACCGCTCCTTTAGGT |
| 38 | Ki67-2 | GCTCGCCTTGATGGTTCCT |
